# Compressing gene expression data using multiple latent space dimensionalities learns complementary biological representations

**DOI:** 10.1186/s13059-020-02021-3

**Published:** 2020-05-11

**Authors:** Gregory P. Way, Michael Zietz, Vincent Rubinetti, Daniel S. Himmelstein, Casey S. Greene

**Affiliations:** 1 Genomics and Computational Biology Graduate Group, Perelman School of Medicine, University of Pennsylvania, Philadelphia, PA 19104 USA; 2 Department of Systems Pharmacology and Translational Therapeutics, University of Pennsylvania, 10-131 SCTR 34th and Civic Center Blvd, Philadelphia, PA 19104 USA; 3 Imaging Platform, Broad Institute of MIT and Harvard, Cambridge, MA 02142 USA; 4 Childhood Cancer Data Lab, Alex’s Lemonade Stand Foundation, Philadelphia, PA 19102 USA

**Keywords:** Machine learning, Dimensionality reduction, Latent space, Gene expression, Autoencoders, Compression, Neural network interpretation

## Abstract

**Background:**

Unsupervised compression algorithms applied to gene expression data extract latent or hidden signals representing technical and biological sources of variation. However, these algorithms require a user to select a biologically appropriate latent space dimensionality. In practice, most researchers fit a single algorithm and latent dimensionality. We sought to determine the extent by which selecting only one fit limits the biological features captured in the latent representations and, consequently, limits what can be discovered with subsequent analyses.

**Results:**

We compress gene expression data from three large datasets consisting of adult normal tissue, adult cancer tissue, and pediatric cancer tissue. We train many different models across a large range of latent space dimensionalities and observe various performance differences. We identify more curated pathway gene sets significantly associated with individual dimensions in denoising autoencoder and variational autoencoder models trained using an intermediate number of latent dimensionalities. Combining compressed features across algorithms and dimensionalities captures the most pathway-associated representations. When trained with different latent dimensionalities, models learn strongly associated and generalizable biological representations including sex, neuroblastoma MYCN amplification, and cell types. Stronger signals, such as tumor type, are best captured in models trained at lower dimensionalities, while more subtle signals such as pathway activity are best identified in models trained with more latent dimensionalities.

**Conclusions:**

There is no single best latent dimensionality or compression algorithm for analyzing gene expression data. Instead, using features derived from different compression models across multiple latent space dimensionalities enhances biological representations.

## Background

Dimensionality reduction algorithms compress input data into feature representations that capture different sources of variation. Applied to gene expression data, compression algorithms can identify latent technical and biological representations. These biological representations reveal important information about the samples and can help to generate hypotheses that are difficult or impossible to observe in the original genomic space. For example, linear methods such as principal component analysis (PCA), independent component analysis (ICA), and non-negative matrix factorization (NMF) have been applied to large transcriptomic compendium to reveal the influence of copy number alterations on gene expression measurements, to identify coordinated transcriptional programs, and to estimate cell-type proportion in bulk tissue samples [1–5]. Additionally, nonlinear methods such as denoising autoencoders (DAE) and variational autoencoders (VAE) have revealed latent signals characterizing oxygen exposure, transcription factor targets, cancer subtypes, and drug response [6–9]. Other latent variable approaches have been used to detect and remove technical artifacts, including batch effects [10, 11]. Here, we focus on using compression algorithms to identify biological representations by analyzing processed data with batch effect already mitigated.

A major challenge to all compression applications is the fundamental requirement that a researcher must determine the number of latent dimensions (*k*) to compress input data. It is possible that different biological representations are best captured using models trained at different latent space dimensionalities. To test this, we trained and evaluated various compression models across a wide range of latent space dimensionalities, from *k* = 2 to *k* = 200. Specifically, we trained PCA, ICA, NMF, DAE, and VAE models using three different gene expression datasets. We selected these methods because they are either widely established in practice (PCA, ICA, NMF) or use neural networks that are rapidly growing in popularity (DAE, VAE). Furthermore, it is well known that PCA will identify a unique and deterministic solution that represents compressed features with a decreasing amount of variance explained [12]. However, the other models do not share this property. In these other models, different latent space dimensionalities and model initializations will identify different feature representations, and the feature number has no inherent ordering [13]. We applied these methods to processed RNAseq data from The Cancer Genome Atlas (TCGA) PanCanAtlas [14], the Genome Tissue Expression Consortium Project (GTEx) [15], and the Therapeutically Applicable Research To Generate Effective Treatments (TARGET) Project [16].

The paper is divided into two parts. First, we describe model performance in different contexts. We observed differences in reconstruction cost, stability, and gene set coverage across datasets, algorithms, and latent dimensionalities. Second, we present a series of vignettes highlighting differences in biological representations driven by the number of latent dimensionalities used during model training. We observed that distinct gene expression representations are best captured in different models spanning low, intermediate, and high latent dimensionalities. Our primary finding is that there is no single algorithm or dimensionality that is best for all purposes: Instead, using various latent dimensionalities and algorithms optimizes biological representations. Researchers who plan to apply these algorithms to gene expression data should consider training multiple models over multiple latent dimensionalities to optimize and avoid missing important biological representations.

We name this multiple compression approach “BioBombe” after the large mechanical device developed by Alan Turing and other cryptologists in World War II to decode encrypted messages sent by Enigma machines. Using the BioBombe approach, we compress gene expression input data using different latent dimensionalities and algorithms to enhance discovery of biological representations. We show that different biological features are best extracted by different models trained with different latent dimensionalities.

## Results

### BioBombe implementation

**Fig. 1:**
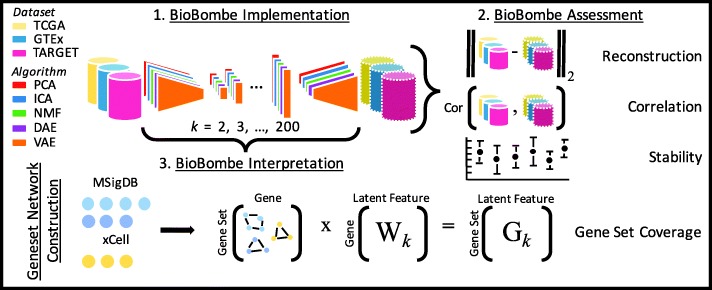
Overview of the BioBombe approach*.* We implemented BioBombe on three datasets using five different algorithms. We compressed input data into various latent dimensionalities. We calculated various metrics that describe different benefits and trade-offs of the algorithms. Lastly, we implemented a network projection approach to interpret the compressed latent features. We used MSigDB collections and xCell gene sets to interpret compressed features

We compressed processed RNAseq data from TCGA, GTEx, and TARGET using PCA, ICA, NMF, DAE, and VAE across 28 different latent dimensionalities (*k*) ranging from *k* = 2 to *k* = 200. We split each dataset into 90% training and 10% test sets balanced by cancer type or tissue type and trained models using only the training data. We used real and permuted data and initialized each model five times per latent dimensionality resulting in a total of 4200 different compression models (Additional file 1: Figure S1). We evaluated hyperparameters for DAE and VAE models across dimensionalities and trained models using optimized parameter settings (Additional file 2; Additional file 1: Figure S2). See Fig. 1 for an outline of our approach. We provide full BioBombe results for all compression models across datasets for both real [17–19] and permuted data [20–22] in both training and test sets as publicly available resources (see https://greenelab.github.io/BioBombe/).

### Assessing compression algorithm reconstruction performance

In the first part of the paper, we report specific and commonly applied performance metrics for all algorithms and latent dimensionalities. Reconstruction cost, a measurement of the difference between the input and output matrices, is often used to describe the ability of compression models to capture fundamental processes in latent space features that recapitulate the original input data. We tracked the reconstruction cost for the training and testing data partitions for all datasets, algorithms, latent dimensionalities, and random initializations. As expected, we observed lower reconstruction costs in models trained with real data and with higher latent dimensionalities (Additional file 1: Figure S3). Because PCA and ICA are rotations of one another, we used their identical scores as a positive control. All the compression algorithms had similar reconstruction costs, with the highest variability existing in low latent dimensionalities (Additional file 1: Figure S3).

### Evaluating model stability and similarity within and across latent dimensionalities

**Fig. 2:**
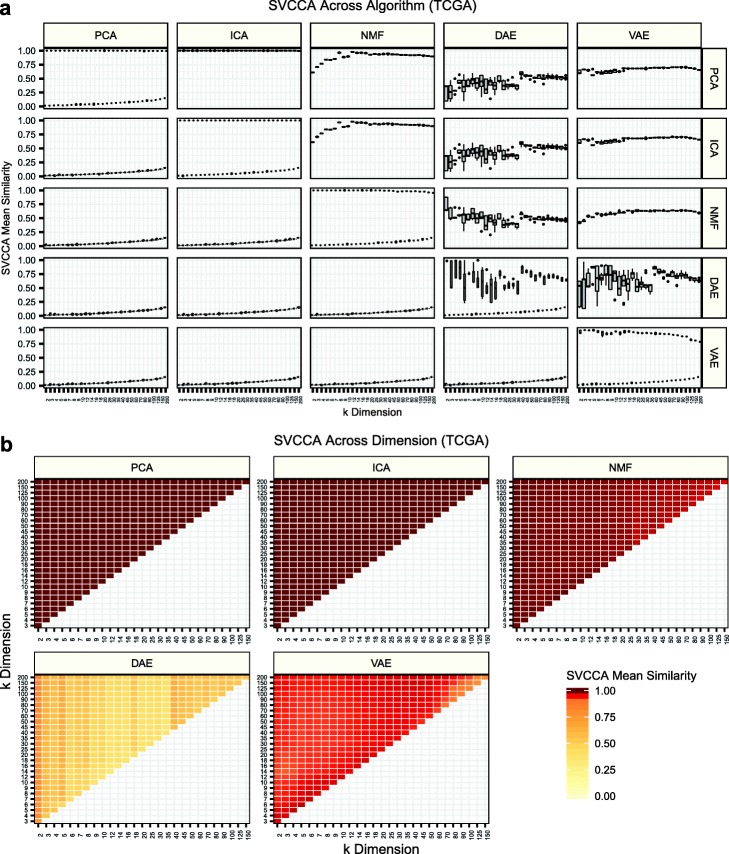
Assessing algorithm and dimensionality stability with singular vector canonical correlation analysis (SVCCA). **a** SVCCA applied to the weight matrices learned by each compression algorithm in gene expression data from The Cancer Genome Atlas (TCGA). The mean of all canonical correlations comparing independent iterations is shown. The distribution of mean similarity represents a comparison of all pairwise iterations within and across algorithms. The upper triangle represents SVCCA applied to real gene expression data, while the lower triangle represents permuted expression data. Both real and permuted data are plotted along the diagonal. **b** Mean correlations of all iterations within algorithms but across *k* dimensionalities. SVCCA will identify min(*i*, *j*) canonical vectors for latent dimensionalities *k*_*i*_ and *k*_*j*_. The mean of all pairwise correlations is shown for all combinations of *k* dimensionalities

We applied singular vector canonical correlation analysis (SVCCA) to algorithm weight matrices to assess model stability within algorithm initializations and to determine model similarity between algorithms [23]. Briefly, SVCCA calculates similarity between two compression algorithm weight matrices by learning appropriate linear transformations and iteratively matching the highest correlating features. Training with TCGA data, we observed highly stable models within algorithms and within all latent dimensionalities for PCA, ICA, and NMF (along the matrix diagonal in Fig. 2a). VAE models were also largely stable, with some decay in higher latent dimensionalities. However, DAE models were unstable, particularly when trained with low latent dimensionalities (Fig. 2a). We also compared similarity across algorithms. Because PCA and ICA are rotations of one another, we used the high stability as a positive control for SVCCA estimates. NMF was also highly similar to PCA and ICA, particularly in models trained with intermediate and high latent dimensionalities (Fig. 2a). VAE models were more similar to PCA, ICA, and NMF than DAE models, particularly at low latent dimensionalities, and the instability patterns within DAE models also led to large differences across algorithms (Fig. 2a). We observed similar patterns in GTEx and TARGET data, despite TARGET containing only about 700 samples (Additional file 1: Figure S4).

We also used SVCCA to compare the similarity of weight matrices extracted from models trained with different latent dimensionalities. Both PCA and ICA found highly similar solutions (Fig. 2b). This is expected since PCA solutions are deterministic and are arranged with decreasing amounts of variance, and ICA is a rotation of PCA space. We do not observe these patterns for the other compression algorithms. While NMF identified highly similar solutions in models trained with low dimensionalities, solutions were less similar in models with higher dimensionalities. DAE solutions were the least similar, with intermediate dimensionality models showing the lowest mean similarity. VAE models displayed relatively high similarity, but there were regions of modest model stability in intermediate and high dimensionalities (Fig. 2b). We observed similar patterns in GTEx and TARGET data (Additional file 1: Figure S5).

### Different latent space dimensionalities and algorithms capture specific gene expression representations at variable resolution

**Fig. 3:**
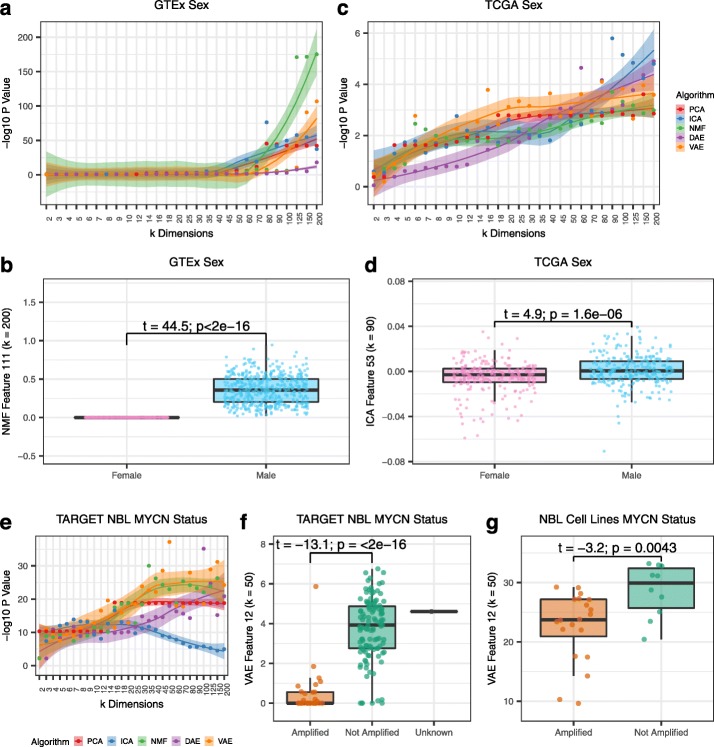
Exploring the ability of BioBombe features across algorithms and latent dimensionalities to detect biological representations. Detecting GTEx sample sex across **a** various latent dimensionalities and algorithms, and **b** the latent feature with the highest enrichment. Detecting TCGA patient sex across **c** various latent dimensionalities, and **d** the latent feature with the highest enrichment. Detecting TARGET MYCN amplification in neuroblastoma (NBL) tumors **e** across various latent dimensionalities, and **f** the latent feature with the highest enrichment. **g** Applying the MYCN representation to an external dataset of NBL cell lines implicates MYCN amplified cell lines

In the second part of the paper, we tested the ability of different latent space dimensionalities and algorithms to capture various biological signals. We first tested the ability of all BioBombe features to differentiate common and well-characterized biological representations. We describe the ability of BioBombe features to isolate sample sex, which has been previously observed to be captured in latent space features [8, 24, 25]. Using BioBombe sample activation scores across all initializations, algorithms, and latent dimensionalities, we performed two-tailed *t*-tests comparing male and female samples in the GTEx test set. Sample activation scores represent the activity of specific samples for a given compressed feature. We identified this phenotype with the highest enrichment in NMF and VAE models trained with higher latent dimensionalities (Fig. 3a). The top feature separating GTEx males and females was NMF feature 111 in *k* = 200 (*t* = 44.5, *p* = 7.3 × 10^− 176^) (Fig. 3b). We examined the genes that contributed with high weight to this feature and found only three genes had substantial influence. These three genes all had high positive weights and were encoded on the Y chromosome. We performed the same approach using BioBombe features to identify sex features in TCGA test data (Fig. 3c). The top latent dimensionality identified was not consistent across algorithms. The top feature distinguishing TCGA males and females was ICA feature 53 in the *k* = 90 model (*t* = 4.9, *p* = 2.0 × 10^− 6^) (Fig. 3d). The separation was not as strong using the more complex TCGA data, but the top 10 gene weights were all encoded on the X chromosome. While this analysis demonstrates that sex is identified with varying signal strength across algorithms and latent dimensionalities, it also highlights that compression algorithms do not completely capture all genes that differentiate sex into a single feature. To identify genes with expression that varies by sex, it would be best to apply a differential expression analysis [26, 27].

We also tested the ability of BioBombe features to distinguish MYCN amplification in neuroblastoma (NBL) tumors. MYCN amplification is a biomarker associated with poor prognosis in NBL patients [28]. Again using all BioBombe sample activation scores, we performed a two-tailed *t*-test comparing MYCN amplified vs. MYCN not amplified NBL tumors in the full set of TARGET samples. Each algorithm best isolated MYCN amplification signal at different latent dimensionalities, but the top scoring features were generally identified in VAE and NMF models trained with large latent spaces (Fig. 3e). Although there were some potentially mischaracterized samples, feature 12 in VAE *k* = 50 robustly separated MYCN amplification status in NBL tumors (*t* = − 18.5, *p* = 6.6 × 10^− 38^) (Fig. 3f). This feature also distinguished MYCN amplification status in NBL cell lines [29] that were previously not used in training the compression model or for feature selection (*t* = − 3.2, *p* = 4.2 × 10^− 3^) (Fig. 3g). Taken together, these analyses demonstrate that different compression models best identify specific biological representations when trained with different latent space dimensionalities.

### Large-scale interpretation of BioBombe compressed features: assessing gene set coverage

The BioBombe approach generates many different features associated with various biological representations. As part of our rigorous evaluation, we generated 30,850 features per dataset. The features are generated in an unsupervised fashion, and, in order to maximize utility, they require interpretation. One interpretation approach involves projecting gene weights onto existing biological networks (see Fig. 1a). This approach not only calculates enrichment scores of specific biological gene sets and pathways for individual BioBombe features, but also enables us to track how these enrichment scores evolve across latent dimensionalities, and to quantify the percentage of characterized gene sets in each collection. We define this percentage as “gene set coverage.”

**Fig. 4:**
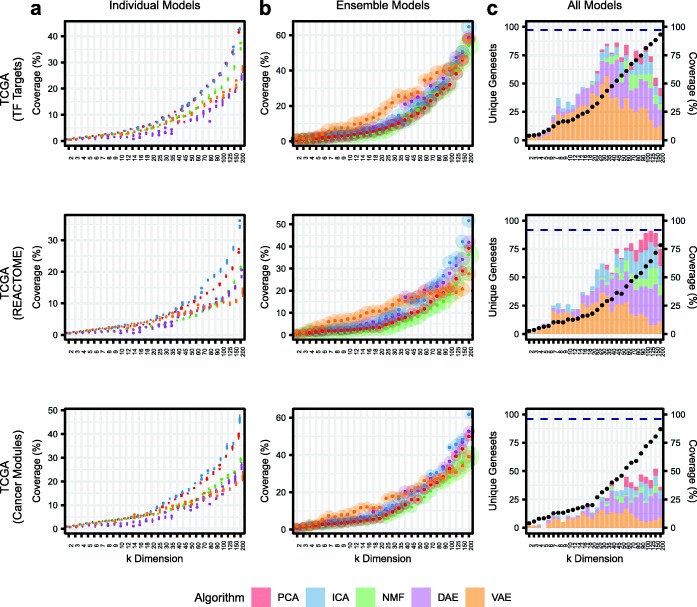
Assessing gene set coverage of specific gene set collections. Tracking results in TCGA data for three gene set collections representing transcription factor (TF) targets (C3TFT), Reactome pathways (C2CPREACTOME), and cancer modules (C4CM). **a** Tracking coverage in individual models, which represents the distribution of scores across five algorithm iterations. **b** Tracking coverage in ensemble models, which represents coverage after combining all five iterations into a single model. The size of the point represents relative enrichment strength. **c** Tracking coverage in all models combined within *k* dimensionalities. The number of algorithm-specific unique gene sets identified is shown as bar charts. Coverage for all models combined across all *k* dimensionalities is shown as a dotted navy blue line

Specifically, we used gene sets from Molecular Signatures Database (MSigDB) and xCell [30–32] to interpret biological signals activated in compressed features across all latent dimensionalities, algorithms, and initializations. We applied a network projection approach to all compression algorithm weight matrices to determine gene set coverage. Briefly, we projected all compressed features onto a gene set network and assigned gene sets with the highest enrichment that passed an adjusted statistical significance threshold to each compressed feature (see “Methods” for more details). We tracked coverage of three MSigDB gene set collections representing transcription factor (TF) targets, cancer modules, and Reactome pathways across latent dimensionalities in TCGA data (Fig. 4). In all cases, and as expected, we observed higher gene set coverage in models trained with larger latent space dimensionalities. Considering individual models, we observed high coverage in PCA, ICA, and NMF. In particular, ICA outperformed all other algorithms (Fig. 4a). However, while these methods showed the highest coverage, the features identified had relatively low enrichment scores compared to AE models potentially indicating that they captured the biological signals to a weaker degree (Additional file 1: Figure S6).

An additional approach to interpreting individual models is to interpret “ensemble” models, which consist of features derived from all five algorithm initializations within each latent dimension (see Additional file 1: Figure S1). Aggregating all five random initializations into ensemble models, we observed substantial coverage increases, especially for AEs (Fig. 4b). This is expected behavior since AE-based models generally have higher instability across initializations, and therefore have more capacity to identify different biological representations. VAE models had high coverage for all gene sets in intermediate dimensions, while DAE improved in higher dimensions. However, at the highest dimensions, ICA still demonstrated the highest coverage. NMF consistently had the highest enrichment scores, but the lowest coverage (Fig. 4b). When considering all models combined (forming an ensemble of algorithm ensembles) within latent dimensionalities, we observed substantially increased coverage of all gene sets. However, most of the unique gene sets were contributed by the AE models (Fig. 4c). Lastly, when we aggregated all BioBombe features across all algorithms and all latent dimensionalities together into a single model, we observed the highest absolute gene set coverage (Fig. 4c). These patterns were consistent across other gene set collections and datasets (Additional file 1: Figure S7). In general, while models compressed with larger latent space dimensionalities had higher gene set coverage, many individual gene sets were captured with the highest enrichment in models with low and intermediate dimensionalities (Additional file 1: Figure S8). These results did not reveal a best method or dimensionality: Various biological representations are best discovered by using various compression algorithms with various latent space dimensionalities.

### Observing strongly associated latent dimensionalities for capturing specific tissue and cell type signals

**Fig. 5:**
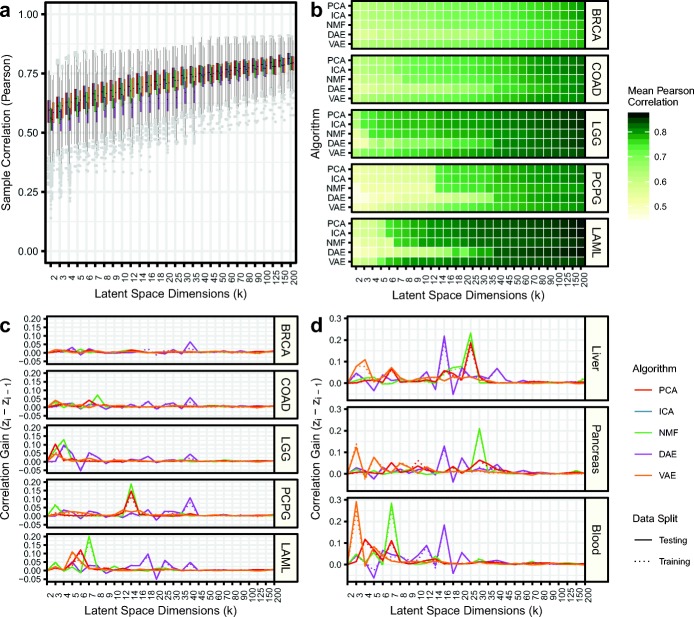
Different latent dimensionalities implicate different tissue types. **a** Sample Pearson correlation for all data in the testing data partition for The Cancer Genome Atlas (TCGA). The different algorithms follow the legend provided in panel d. **b** Mean Pearson correlation for select cancer types in the testing data partition. Pearson correlation gain between sequential latent dimensionalities for **c** select cancer types in TCGA and **d** select tissue types in GTEx

Next, we closely tracked the ability of compression models to capture specific information about sample composition across latent dimensionalities. We measured the Pearson correlation between all samples’ gene expression input and reconstructed output. Like reconstruction, we use sample correlation to determine how well the compressed features describe the given sample. Overall, we observed increased mean correlation and decreased variance as the latent dimensionalities increased in TCGA data (Fig. 5a). We also observed similar patterns in GTEx and TARGET data (Additional file 1: Figure S9). Correlation was not consistent across algorithms as PCA, ICA, and NMF generally outperformed the AE models. Across all datasets, in randomly permuted data, we observed correlations near zero (Additional file 1: Figure S9).

Correlation with reconstructed output can be measured for individual samples. We tracked correlation across latent dimensionalities to determine which latent feature captures specific tissue types. In most cases, we observed small increases in sample correlation with increasing latent dimensionality. For example, breast-invasive carcinoma (BRCA) and colon adenocarcinoma (COAD) displayed relatively gradual increases in sample correlation as the latent dimensionality increased (Fig. 5b). However, in other cancer types, such as low-grade glioma (LGG), pheochromocytoma and paraganglioma (PCPG), and acute myeloid leukemia (LAML), we observed large correlation gains with a single increase in latent dimensionality (Fig. 5c). We also observed similar performance spikes in GTEx data for several tissues including liver, pancreas, and blood (Fig. 5d). This sudden and rapid increase in correlation in specific tissues occurred at different latent dimensionalities for different algorithms, but was consistent across algorithm initializations.

**Fig. 6:**
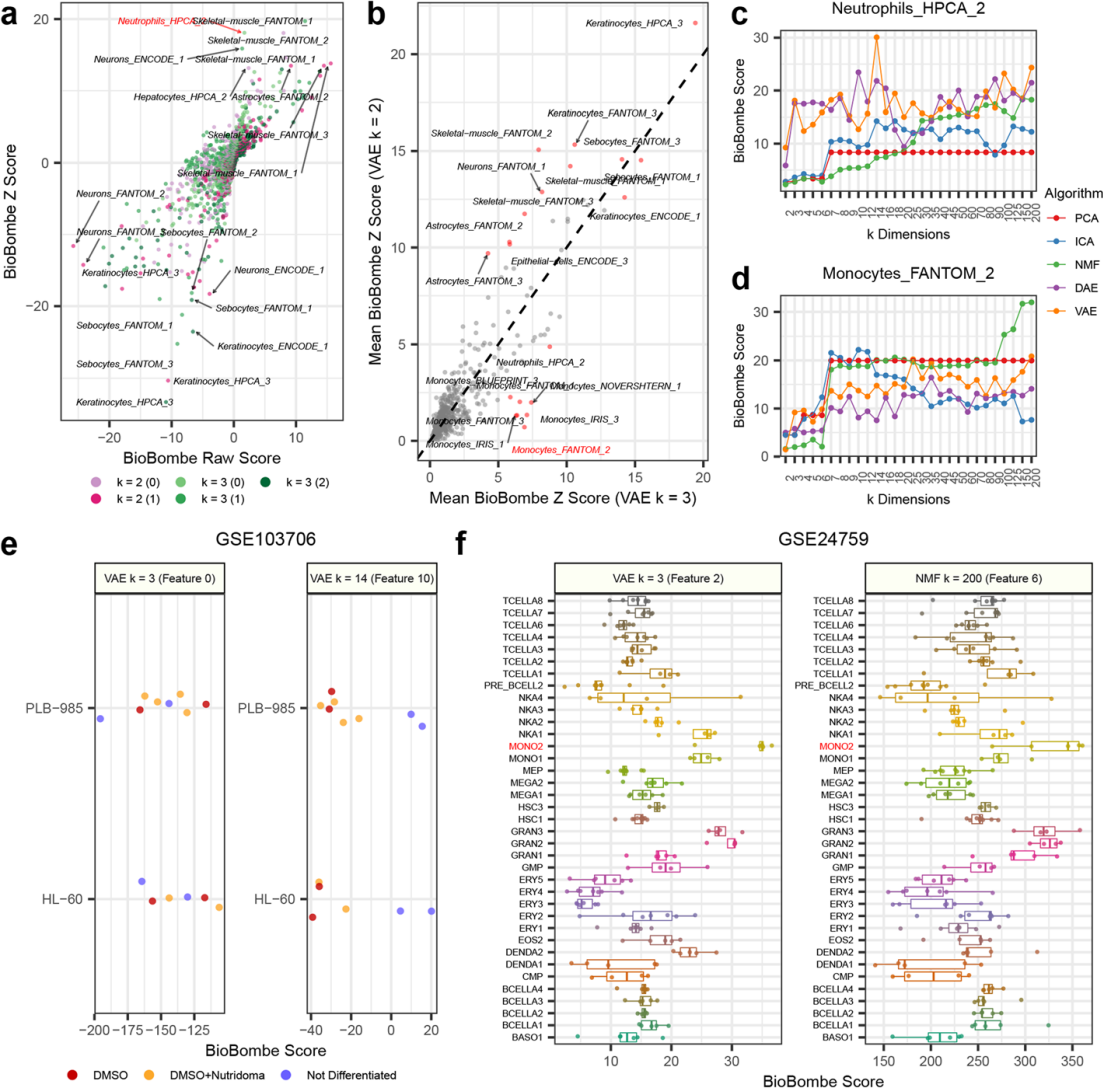
Interpreting blood cell types in GTEx using xCell gene sets. **a** Comparing BioBombe scores of all compressed latent features for variational autoencoder (VAE) models when bottleneck dimensionalities are set to *k* = 2 and *k* = 3. **b** Comparing mean BioBombe *z*-scores of aggregated latent features across two VAE models with *k* dimensionalities 2 and 3. Tracking the BioBombe *z*-scores of **c** “Neutrophils_HPCA_2” and **d** “Monocytes_FANTOM_2” gene sets across dimensionalities and algorithms. Only the top scoring feature per algorithm and dimensionality is shown. **e** Projecting the VAE feature *k* = 3 feature and the highest scoring feature (VAE *k* = 14) that best captures a neutrophil representation to an external dataset measuring neutrophil differentiation treatments (GSE103706). **f** Projecting the VAE *k* = 3 feature that best captures monocytes and the feature of the top scoring model (NMF *k* = 200) to an external dataset of isolated hematopoietic cell types (GSE24759)

To determine if this rapid increase was a result of models learning specific biological representations or if this observation represented a technical artifact, we more closely examined the sharp increase in GTEx blood tissue correlation between latent space dimensionalities 2 and 3 in VAE models (See Fig. 5d). We hypothesized that a difference in sample correlation for a specific tissue at such a low dimensionality could be driven by a change in the cell types captured by the model. We applied network projection of xCell gene sets to all compressed features in both VAE models. xCell gene sets represent computationally derived cell type signatures [31]. The top features identified for the VAE *k* = 2 model included skeletal muscle, keratinocyte, and neuronal gene sets (Fig. 6a). Skeletal muscle was the most significant gene set identified likely because it is the tissue with the most samples in GTEx. Similar gene sets were enriched in the *k* = 3 model, but we also observed enrichment for a specific neutrophil gene set (“Neutrophils_HPCA_2”) (Fig. 6a). Neutrophils represent 50% of all blood cell types, which may explain the increased correlation in blood tissue observed in VAE *k* = 3 models. The features implicated using the network projection approach were similar to an overrepresentation analysis using high weight genes in both tails of the VAE *k* = 3 feature (Additional file 1: Figure S10).

We also calculated the mean absolute value *z*-scores for xCell gene sets in all compression features for both VAE models with *k* = 2 and *k* = 3 dimensionalities (Fig. 6b). Again, we observed skeletal muscle, keratinocytes, and neuronal gene sets to be enriched in both models. However, we also observed a cluster of monocyte gene sets (including “Monocytes_FANTOM_2”) with enrichment in *k* = 3, but low enrichment in *k* = 2 (Fig. 6b). Monocytes are also important cell types found in blood, and it is probable these signals also contributed to the increased correlation for the reconstructed blood samples in VAE *k* = 3 models. We provide the full list of xCell gene set genes for the neutrophil and monocyte gene sets that intersected with the GTEx data in Additional file 3.

We scanned all other algorithms and latent dimensionalities to identify other compression features with high enrichment scores in the “Neutrophils_HPCA_2” (Fig. 6c) and “Monocytes_FANTOM_2” gene sets (Fig. 6d). We observed stronger enrichment of the “Neutrophil_HPCA_2” gene set in AE models compared to PCA, ICA, and NMF, especially at lower latent dimensionalities. In addition to observing sharp increases in score between VAE *k* = 2 and VAE *k* = 3 models, we also observed that VAE *k* = 14 models produced the highest score for the “Neutrophil_HPCA_2” gene set (Fig. 6c). The top VAE feature at *k* = 14 correlated strongly with the VAE feature learned at *k* = 3 (Additional file 1: Figure S10). Conversely, PCA, ICA, and NMF identified the “Monocytes_FANTOM_2” signature with higher enrichment than the AE models (Fig. 6d). We observed a performance spike at *k* = 7 for both PCA and NMF models, but the highest enrichment for “Monocytes_FANTOM_2” occurred at *k* = 200 in NMF models.

Figure 6 c and d provide concrete examples of differences across algorithm and latent space dimensionalities. Specifically, PCA identifies both neutrophil and monocyte gene sets at *k* = 6, and then does not identify any other feature with more signal. This is expected since PCA solutions are deterministic and ordered by decreasing variance explained. However, we do not observe this pattern in other algorithms. The AE models capture the neutrophil representation early and then fluctuate in isolating its signal (Fig. 6c). Furthermore, while all algorithms capture monocytes around *k* = 6, NMF increases signal capture in larger latent dimensionality models and ICA decreases signal capture. Lastly, PCA captures this monocyte gene set as well as other algorithms for most dimensionalities, but the opposite is true for the neutrophil gene set. A researcher selecting only a single compression model at one latent space dimensionality might clearly observe certain features while others could be obscured.

### Validating neutrophil and monocyte representations in external datasets

In order to demonstrate that these neutrophil and monocyte features represent real biology, we applied them to two external datasets that captured each signal using unique experiments. We downloaded a processed gene expression dataset (GSE103706) that applied two treatments to induce neutrophil differentiation in two leukemia cell lines [33]. We hypothesized that projecting the dataset on the “Neutrophil_HPCA_2” signature would reveal differential scores in the treated cell lines. We observed large differences in sample activations of treated vs untreated cell lines in the top neutrophil representation (VAE *k* = 14) (Fig. 6e). We also tested the “Monocytes_FANTOM_2” signature on a different publicly available dataset (GSE24759) measuring gene expression of isolated cell types undergoing hematopoiesis [34]. We observed increased scores for an isolated monocyte cell population (MONO2) and relatively low scores for several other cell types for implicated VAE and top NMF features (Fig. 6f). We observed variable enrichment patterns across different algorithms and latent dimensionalities (Additional file 1: Figure S11a). These separation patterns were associated with network projection scores in NMF models, but were not consistent with other algorithms (Additional file 1: Figure S11b). Taken together, in this analysis, we determined that (1) adding a single latent dimensionality that captured neutrophils and monocytes improved signal detection in GTEx blood, (2) these cell-type representations are enhanced at different latent dimensionalities and by different algorithms, and (3) these representations generalized to external datasets that were not encountered during model training.

### Detecting both strong and subtle signals by compressing gene expression data

**Fig. 7:**
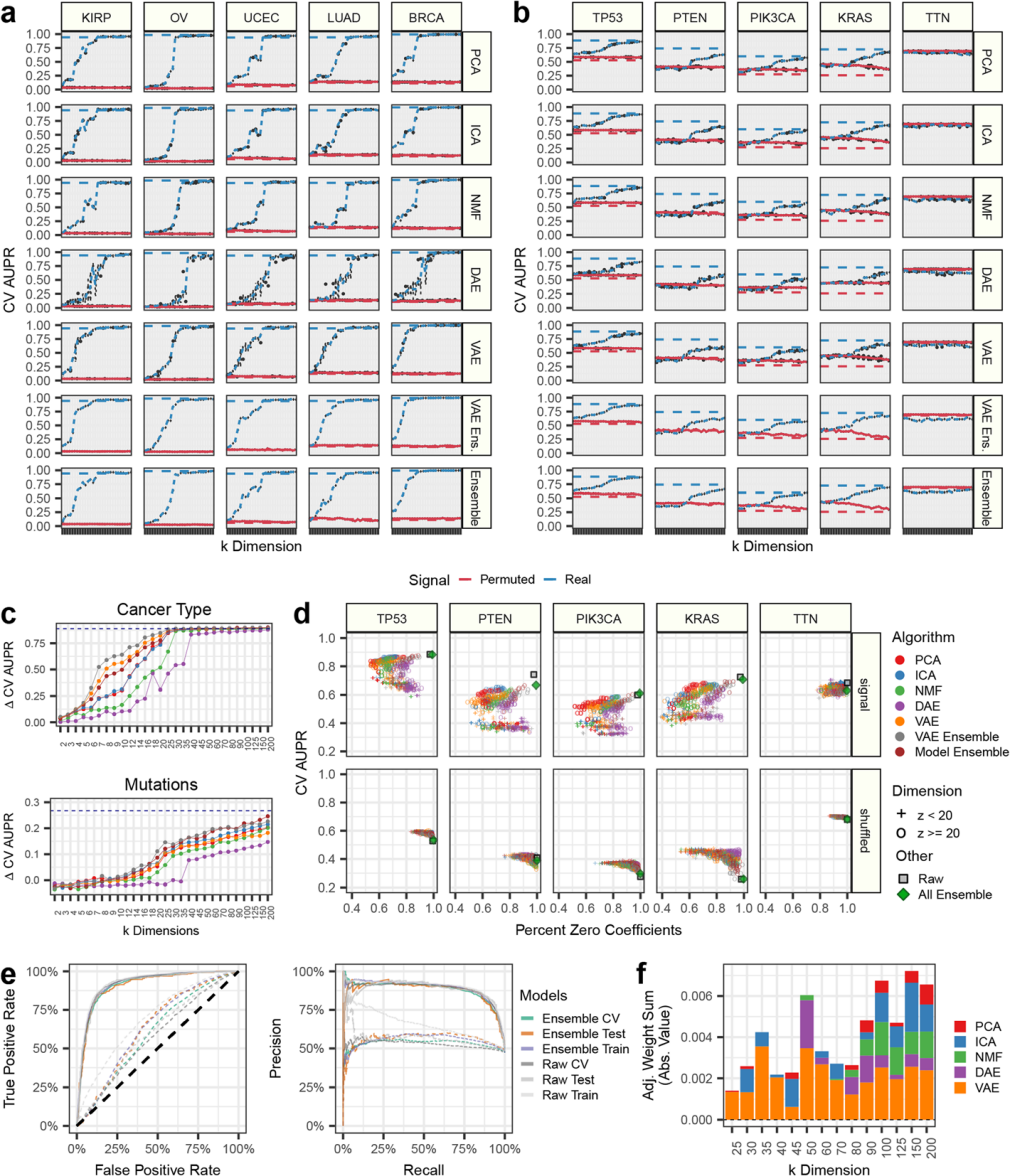
Using BioBombe compressed features as input in supervised machine learning tasks. Predicting **a** cancer type status and **b** gene mutation status for select cancer types and important cancer genes using five compression algorithms and two ensemble models in TCGA data. The area under the precision recall (AUPR) curve for cross validation (CV) data partitions is shown. The blue lines represent predictions made with permuted data input into each compression algorithm. The dotted lines represent AUPR on untransformed RNAseq data. The dotted gray line represents a hypothetical random guess. **c** Tracking the average change in AUPR between real and permuted data across latent dimensionalities and compression models in predicting (*top*) cancer types and (*bottom*) mutation status. The average includes the five cancer types and mutations tracked in panels **a** and **b**. **d** Tracking the sparsity and performance of supervised models using BioBombe compressed features in real and permuted data. **e** Performance metrics for the all-compression feature ensemble model predicting *TP53* alterations. (*left*) Receiver operating characteristic (ROC) and (*right*) precision recall curves are shown. **f** The average absolute value weight per algorithm for the all-compression-feature ensemble model predicting *TP53* alterations. The adjusted scores are acquired by dividing by the number of latent dimensionalities in the given model

We tested the ability of BioBombe features to capture cancer type and genetic alterations in two distinct supervised machine learning experiments. Cancer-type represents a strong signal, while genetic alterations are typically subtle [35]. In both experiments, we trained logistic regression models with an elastic net penalty using compressed BioBombe features as input. First, we trained models to predict each of the 33 different TCGA cancer types. Using BioBombe features across algorithms and latent dimensionalities, nearly all cancer types could be predicted with high precision and recall (Additional file 1: Figure S12). We observed multiple performance spikes at varying latent dimensionalities for different cancer types and algorithms, which typically occurred in small latent dimensionalities (Fig. 7a). Next, we trained models to predict alterations in the top 50 most mutated genes in TCGA (Additional file 1: Figure S13). We focused on predicting four cancer genes and one negative control; *TP53*, *PTEN*, *PIK3CA*, *KRAS*, and *TTN* (Fig. 7b). *TTN* is a particularly large gene and is associated with a high passenger mutation burden and should provide no predictive signal [36]. As expected, we did not observe any signal in predicting *TTN* (Fig. 7b). Again, we observed performance increases at varying latent dimensionalities across algorithms. However, predictive signal for mutations occurred at higher latent dimensionalities compared to cancer types (Fig. 7c). This result suggests that more subtle features are captured only when a compression algorithm is provided enough latent dimensions to describe the signal. Compared to features trained within algorithm and within iteration, an ensemble of five VAE models and an ensemble of five models representing one iteration of each algorithm (PCA, ICA, NMF, DAE, and VAE) identified cancer type and mutation status in earlier dimensionalities compared to single model iterations (Fig. 7c). We also tracked the logistic regression coefficients assigned to each compression feature. DAE models consistently displayed sparse models, and the VAE ensemble and model ensemble also induced high sparsity (Fig. 7d).

Lastly, we trained logistic regression classifiers using all 30,850 BioBombe features generated across iterations, algorithms, and latent dimensionalities. These models were sparse and high performing, comparable to logistic regression models trained using raw features (Fig. 7e). Of all 30,850 compressed features in the model predicting TP53 alterations, only 317 were assigned non-zero weights (1.03%). We applied the network projection approach using Hallmark gene sets to interpret the biological signals of the top supervised model coefficients. The top positive feature was derived from a VAE trained with *k* = 200. The top hallmarks of this feature included “ESTROGEN_RESPONSE_EARLY,” “ESTROGEN_RESPONSE_LATE,” and “P53_PATHWAY.” The top negative feature was derived from a VAE trained with *k* = 150 and was associated with hallmark gene sets including “BILE_ACID_METABOLISM,” “EPITHELIAL_MESENCHYMAL_TRANSITION,” and “FATTY_ACID_METABOLISM.” Additional file 4 includes a full list of logistic regression coefficients and hallmark network projection scores. Overall, the features selected by the supervised classifier were distributed across algorithms and latent dimensionalities suggesting that combining compression features across dimensionalities and algorithms provided the best representation of the signal (Fig. 7f).

## Discussion

Our primary observation is that compressing complex gene expression data using multiple latent dimensionalities and algorithms improves discovery of biological representations. Across different latent dimensionalities and algorithms, we identified optimal features to stratify sample sex, MYCN amplification, blood cell types, cancer types, and mutation status. These features generalized to other data, providing additional evidence for the intrinsic qualities of biological representations embedded in gene expression data [25, 37–39]. Furthermore, the complexity of biological features was associated with the number of latent dimensionalities used. We predicted gene mutation using models with high dimensionality, but we detected cancer type with high accuracy using models with low dimensionality. In general, unsupervised learning algorithms applied to gene expression data extract biological and technical signals present in input samples. However, both the latent dimensionality and algorithm selected contribute strongly to the biological representations that are identified.

When applying these algorithms, researchers must determine how many latent dimensions to compress their input data into and different studies can have a variety of goals. For example, compression algorithms used for visualization can stratify sample groups based on the largest sources of variation [40–45]. In visualization settings, selecting a small number of latent dimensions is often best, and there is no need to compress data across multiple latent dimensionalities. However, if the analysis goal includes learning biological representations to identify more subtle patterns in input samples, then there is not a single optimal latent dimensionality nor optimal algorithm. For example, though ICA and PCA represent rotations of each other, we found that the methods varied in their ability to capture specific biological signals into single features, which highlights the challenge of picking only a single algorithm. While compressing data into a single latent dimensionality will capture many biological signals, the “correct” dimensionality is not always clear, and several biological representations may be better revealed by alternative latent dimensionalities.

If optimizing a single model, a researcher can use one or many criteria to select an appropriate latent dimensionality. Measurements such as Akaike information criterion (AIC), Bayesian information criterion (BIC), stability, and cross validation (CV) can be applied to a series of latent dimensionalities [13, 46, 47]. Other algorithms, like Dirichlet processes, can naturally arrive at an appropriate dimensionality through several algorithm iterations [48]. Hidden layer dimensions of unsupervised neural networks are tunable hyperparameters defined by expected input data complexity and performance. However, applied to gene expression data, these metrics often provide conflicting results and unclear suggestions. In genomics applications, the method Thresher uses a combination of outlier detection and PCA to identify the optimal number of clusters [49]. Compression model stability can also be used to determine an optimal latent dimensionality in gene expression data [50]. By considering only reproducible features, ICA revealed 139 modules from nearly 100,000 publicly available gene expression profiles [51]. However, we argue that these metrics can be misleading. Rather than using heuristics to select a biologically appropriate latent dimensionality, a researcher may instead elect to compress gene expression data into many different latent space dimensionalities to generate many different feature representations.

There are many limitations in our evaluation. First, our approach takes a long time to run. We are training many different algorithms across many different latent dimensionalities and iterations, which requires a lot of compute time (Additional file 1: Figure S14). However, because we are training many models independently, this task can be parallelized. Additionally, we did not evaluate dimensionalities above *k* = 200. It is likely that many more representations can be learned, and possibly with even higher association strengths in higher dimensionalities for certain biology. We also did not focus on detecting compressed features that represent technical artifacts, which has already been widely explored [52, 53]. Furthermore, the BioBombe approach is not a replacement for differential gene expression analysis in implicating genes associated with a specific phenotype. For example, if a scientist’s goal was to identify all genes contributing to sex differences or MYCN amplification, then they would apply a differential expression analysis. We used these vignettes to demonstrate trade-offs in how well different algorithms and latent dimensionalities capture these signals. Moreover, we did not explore adding hidden layers in AE models. Many models trained on gene expression data have benefited from using multiple hidden layers in neural network architectures [7, 54]. Additional methods, like DeepLift, can be used to reveal gene importance values in internal representations of deep networks [55, 56].

An additional challenge is interpreting the biological content of the compressed gene expression features. Overrepresentation analysis (ORA) and gene set enrichment analysis (GSEA) are commonly applied but have significant limitations [30, 57]. ORA requires a user to select a cutoff, typically based on standard deviation, to build representative gene sets from each feature. ORA tests also do not consider the weights, or gene importance scores, in each compression feature. Conversely, GSEA operates on ranked features, but often requires many permutations to establish significance. Furthermore, ORA requires each tail of the compressed feature distribution to be interpreted separately in algorithms that also learn negative weights. The weight distribution is dependent on the specific compression algorithm, and the same cutoff may not be appropriate for all algorithms and all compressed features. Here, we present a novel option to rapidly interpret compressed features based on network projection [58, 59]. The approach is applied to the full and continuous distribution of gene weights, operates independently of the algorithm feature distribution, does not require arbitrary thresholds, and obviates the need to consider both tails of the distribution separately. Nevertheless, additional downstream experimental validation is necessary to determine if the constructed feature actually represents the biology it has been assigned.

## Conclusions

To enhance biological representations discovered in a given dataset, it is best to compress gene expression data using several algorithms and many different latent space dimensionalities. These compressed gene expression features represent important biological signals, including various cell types, phenotypes, biomarkers, and other sample characteristics. We showed, through several experiments tracking lower dimensional gene expression representations, gene set coverage, and supervised learning performance, that optimal biological features are learned using a variety of latent space dimensionalities and different compression algorithms. As unsupervised machine learning continues to be applied to derive insight from biomedical datasets, researchers should shift focus away from optimizing a single model based on certain mathematical heuristics, and instead towards learning good and reproducible biological representations that generalize to alternative datasets regardless of compression algorithm and latent dimensionality.

## Methods

### Transcriptomic compendia acquisition and processing

We downloaded transcriptomic datasets from publicly available resources. We downloaded the batch-corrected TCGA PanCanAtlas RNAseq data from the National Cancer Institute Genomic Data Commons (https://gdc.cancer.gov/about-data/publications/pancanatlas). These data consisted of 11,069 samples with 20,531 measured genes quantified with RSEM and normalized with log transformation. We converted Hugo Symbol gene identifiers into Entrez gene identifiers and discarded non-protein-coding genes and genes that failed to map. We also removed tumors that were measured from multiple sites. This resulted in a final TCGA PanCanAtlas gene expression matrix with 11,060 samples, which included 33 different cancer types, and 16,148 genes. The breakdown of TCGA samples by cancer type is provided in Additional file 5.

We downloaded the TPM normalized GTEx RNAseq data (version 7) from the GTEx data portal (https://gtexportal.org/home/datasets). There were 11,688 samples and 56,202 genes in this dataset. After selecting only protein-coding genes and converting Hugo Symbols to Entrez gene identifiers, we considered 18,356 genes. There are 53 different detailed tissue types in this GTEx version. The tissues types included in these data are provided in Additional file 5.

Lastly, we retrieved the TARGET RNAseq gene expression data from the UCSC Xena data portal [60]. The TARGET data was processed through the FPKM UCSC Toil RNAseq pipeline and was normalized with RSEM and log transformed [61]. The original matrix consists of 734 samples and 60,498 Ensembl gene identifiers. We converted the Ensembl gene identifiers to Entrez gene names and retained only protein-coding genes. This procedure resulted in a total of 18,753 genes measured in TARGET. There are 7 cancer types profiled in TARGET and the specific breakdown is available in Additional file 5. All specific downloading and processing steps can be viewed and reproduced at https://github.com/greenelab/BioBombe/tree/master/0.expression-download.

### Training unsupervised neural networks

Autoencoders (AE) are unsupervised neural networks that learn through minimizing the reconstruction of input data after passing the data through one or several intermediate layers [62]. Typically, these layers are of a lower dimensionality than the input, so the algorithms must compress the input data. Denoising autoencoders (DAE) add noise to input layers during training to regularize solutions and improve generalizability [63]. Variational autoencoders (VAE) add regularization through an additional penalty term imposed on the objective function [64, 65]. In a VAE, the latent space dimensions (*k*) are penalized with a Kullback-Leibler (KL) divergence penalty restricting the distribution of samples in the latent space to Gaussian distributions. We independently optimized each AE model across a grid of hyperparameter combinations including six representative latent dimensionalities (described in Additional file 2 and Additional file 1: Figure S2).

### Training compression algorithms across latent dimensionalities

Independently for each dataset (TCGA, GTEx, and TARGET), we performed the following procedure to train the compression algorithms. First, we randomly split data into 90% training and 10% testing partitions. We balanced each partition by cancer type or tissue type, which meant that each split contained relatively equal representation of tissues. Before input into the compression algorithms, we transformed the gene expression values by gene to the [0, 1] range by subtracting the minimum value and dividing by the range for each specific gene. We applied this transform independently for the testing and training partitions. We selected this range because it was compatible with all of the algorithms. We used the training set to train each compression algorithm. We used the scikit-learn implementations of PCA, ICA, and NMF, and the Tybalt implementations of VAE and DAE [8, 66].

After learning optimized compression models with the training data, we transformed the testing data using these models. We assessed performance metrics using both training and testing data to reduce bias. In addition to training with real data, we also trained all models with randomly permuted data. To permute the training data, we randomly shuffled the gene expression values for all genes independently. We also transformed testing partition data with models trained using randomly permuted data. Training with permuted data removes the correlational structure in the data and can help set performance metric baselines.

One of our goals was to assess differences in performance and biological signal detection across a range of latent dimensionalities (*k*). To this end, we trained all algorithms with various *k* dimensionalities including *k* = 2, 3, 4, 5, 6, 7, 8, 9, 10, 12, 14, 16, 18, 20, 25, 30, 35, 40, 45, 50, 60, 70, 80, 90, 100, 125, 150, and 200 for a total of 28 different dimensionalities. All of these models were trained independently. Lastly, for each *k* dimensionality, we trained five different models initialized with five different random seeds. In total, considering the three datasets, five algorithms, randomly permuted training data, all 28 *k* dimensionalities, and five initializations, we trained 4200 different compression models (Additional file 2: Figure S1). Therefore, in total, we generated 185,100 different compression features.

### Evaluating compression algorithm performance

We evaluated all compression algorithms on three main tasks: reconstruction, sample correlation, and weight matrix stability. First, we evaluated how well the input data is reconstructed after passing through the bottleneck layer. Because the input data was transformed to a distribution between 0 and 1, we used binary cross entropy to measure the difference between algorithm input and output as a measure of reconstruction cost. The lower the reconstruction cost, the higher fidelity reconstruction, and therefore the higher proportion of signals captured in the latent space features. We also assessed the Pearson correlation of all samples comparing input to reconstructed output. This value is similar to reconstruction and can be quickly tracked at an individual sample level. Lastly, we used singular vector canonical correlation analysis (SVCCA) to determine model stability within and model similarity between algorithms and across latent dimensionalities [23]. The SVCCA method consisted of two distinct steps. First, singular value decomposition (SVD) was performed on two input weight matrices. The singular values that combined to reconstruct 98% of the signal in the data were retained. Next, the SVD transformed weight matrix was input into a canonical correlation analysis (CCA). CCA aligned different features in the weight matrix based on maximal correlation after learning a series of linear transformations. Taken together, SVCCA outputs a single metric comparing two input weight matrices that represents stability across model initializations and average similarity of two different models. Because we used the weight matrices, the similarity describes gene expression representation discovery. We use the distribution of SVCCA similarity measures across all pairwise algorithm initializations and latent dimensionalities to indicate model stability [23].

### Assessing gene expression representations present in BioBombe features

We tested BioBombe sequentially compressed features to distinguish sample sex in GTEx and TCGA data, and MYCN amplification in TARGET NBL data. We tested all compression algorithms and latent space dimensionalities to determine the conditions in which these features were best captured. First, we selected tissue types and cancer types in the GTEx and TCGA sex analyses that were balanced by sex by selecting tissues with male to female ratios between 0.5 and 1.5. We performed a two-tailed independent *t*-test assuming unequal variance comparing male and female samples, and NBL samples with and without MYCN amplification. We applied the *t*-test to all compression features identified across algorithms, initializations, and dimensionalities. Shown in the figures are the top scoring feature per latent space dimensionality and algorithm.

We applied the optimal MYCN representation learned in TARGET to an alternative dataset consisting of a series of publicly available NBL cell lines [29]. The data were processed using STAR, and we accessed the processed FPKM matrix from figshare [67]. We transformed the dataset with the identified representations using the following operation:

$$ {R}_{g\prime}^T\ast {D}_{g\prime \mathrm{x}\ n}={D}_{r\ x\ n}^{\prime } $$

where *D* represents the respective RNAseq data to transform, *R* represents the specific compressed feature representation, *g’* represents the overlapping genes measured in both datasets, *n* represents samples, and *D’*_*r*_ represents the compression feature scores in the transformed dataset. Of the 8000 genes measured in TARGET data, 7653 were also measured in external NBL cell line dataset (95.6%).

Using the sample activation scores for each of the top scoring features for sample sex in TCGA and GTEx, and MYCN amplification in TARGET and the validation set, we performed two-tailed *t*-test with unequal variance comparing each group. For the TCGA and GTEx sex comparison, our *t*-test compared male vs. female activation scores. For the TARGET and NBL cell line analyses, our *t*-test compared MYCN amplified NBL samples vs. MYCN non-amplified NBL samples. We add *t*-test statistics and *p* values in each subfigure.

### Gene network construction and processing

We constructed networks using gene set collections compiled by version 6.2 of the Molecular Signatures Database (MSigDB) and cell types derived from xCell [30–32]. These gene sets represent a series of genes that are involved in specific biological processes and functions. We integrated all openly licensed MSigDB collections which included hallmark gene sets (H), positional gene sets (C1), curated gene sets (C2), motif gene sets (C3), computational gene sets (C4), Gene Ontology (GO) terms (C5), oncogenic gene sets (C6), and immunologic gene sets (C7). We omitted MSigDB gene sets that were not available under an open license (KEGG, BioCarta, and AAAS/STKE). The C2 gene set database was split into chemical and genetic perturbations (C2.CPG) and Reactome (C2.CP.Reactome). The C3 gene set was split into microRNA targets (C3.MIR) and transcription factor targets (C3.TFT). The C4 gene set was split into cancer gene neighborhoods (C4.CGN) and cancer modules (C4.CM). Lastly, the C5 gene set was split into GO Biological Processes (C5.BP), GO Cellular Components (C5.CC), and GO molecular functions (C5.MF). xCell represents a gene set compendia of 489 computationally derived gene signatures from 64 different human cell types. The number of gene sets in each curation is provided in Additional file 6. In BioBombe network projection, only a single collection is projected at a time.

To build the gene set network, we used hetnetpy [68]. Briefly, hetnetpy builds networks that include multiple node types and edge relationships. We used hetnetpy to build a single network containing all MSigDB collections and xCell gene sets listed above. The network consisted of 17,451 unique gene sets and 2,159,021 edges representing gene set membership among 20,703 unique gene nodes (Additional file 6). In addition to generating a single network using curated gene sets, we also used hetnetpy to generate 10 permuted networks. The networks are permuted using the XSwap algorithm, which randomizes connections while preserving node degree (i.e., the number of gene set relationships per gene) [69]. Therefore, the permuted networks are used to control for biases induced by uneven gene degree. We compared the observed score against the distribution of permuted network scores to interpret the biological representations in each compression feature.

### Rapid interpretation of compressed gene expression data

Our goal was to quickly interpret the automatically generated compressed latent features learned by each unsupervised algorithm. To this end, we constructed gene set adjacency matrices with specific MSigDB or xCell gene set collections using hetnetpy software. We then performed the following matrix multiplication against a given compressed weight matrix to obtain a raw score for all gene sets for each latent feature.

$$ {H}_{c\ \mathrm{x}\ n}\times {W}_{n\ \mathrm{x}\ k}={G}_{c\ \mathrm{x}\ k} $$

where *H* represents the gene set adjacency matrix, *c* is the specific gene set collection, and *n* represents genes. *W* represents the specific compression algorithm weight matrix, which includes *n* genes and *k* latent space features. The output of this matrix multiplication, *G*, is represented by *c* gene sets and *k* latent dimensions. Through a single matrix multiplication, the matrix *G* tracks raw BioBombe scores.

Because certain hub genes are more likely to be implicated in gene sets and longer gene sets will receive higher raw scores, we compared *G* to the distribution of permuted scores against all 10 permuted networks.

$$ {H_p}_{c\ \mathrm{x}\ n}^{1-10}\times {W}_{n\ \mathrm{x}\ k}={G}_p $$

$$ {G}_{z-\mathrm{score}}=\frac{G_{c\ \mathrm{x}\ k}-\overline{G_p\ }}{\sigma \left({G}_p\right)} $$

where *H*_*P*_^*1–10*^ represents the adjacency matrices for all 10 permuted networks and *G*_*p*_ represents the distribution of scores for the same *k* features for all permutations. We calculated the *z*-score for all gene sets by latent features (*G*_*z-*score_). This score represents the BioBombe Score. Other network-based gene set methods consider gene set influence based on network connectivity of gene set genes [58, 59]. Instead, we used the latent feature weights derived from unsupervised compression algorithms as input, and the compiled gene set networks to assign biological function.

We also compared the BioBombe network projection approach to overrepresentation analyses (ORA). We did not compare the approach to gene set enrichment analysis (GSEA) because evaluating single latent features required many permutations and did not scale to the many thousands of compressed features we examined. We implemented ORA analysis using Fisher’s exact test. The background genes used in the test included only the genes represented in the specific gene set collection.

### Calculating gene set coverage across BioBombe features

We were interested in determining the proportion of gene sets within gene set collections that were captured by the features derived from various compression algorithms. We considered a gene set “captured” by a compression feature if it had the highest positive or highest negative BioBombe *z*-score compared to all other gene sets in that collection. We converted BioBombe *z*-scores into *p* values using the pnorm() R function using a two-tailed test. We removed gene sets from consideration if their *p* values were not lower than a Bonferroni adjusted value determined by the total number of latent dimensionalities in the model.

We calculated coverage (C) by considering all unique top gene sets (*U*) identified by all features in the compression model (*w*) and dividing by the total number of gene sets in the collection (*T*_*C*_).

$$ C=\frac{U_w}{T_c} $$

We calculated the coverage metric for all models independently (C_*i*_), for ensembles, or individual algorithms across all five iterations (*C*_*e*_), and for all models across *k* dimensions (*C*_*k*_).

We also calculated the total coverage of all BioBombe features combined in a single model (*C*_all_). A larger coverage value indicated a model that captured a larger proportion of the signatures present in the given gene set collection.

### Downloading and processing publicly available expression data for neutrophil GTEx analysis

We used an external dataset to validate the neutrophil feature learned by compressing GTEx gene expression data into three latent dimensionalities. We observed that this feature contributed to improved reconstruction of blood tissue. To assess the performance of this neutrophil representation, we downloaded data from the Gene Expression Omnibus (GEO) with accession number GSE103706 [33]. RNA was captured in this dataset using Illumina NextSeq 500. The dataset measured the gene expression of several replicates of two neutrophil-like cell lines, HL-60 and PLB-985, which were originally derived from acute myeloid leukemia (AML) patients. The PLB-985 cell line was previously identified as a subclone of HL-60, so we expect similar activity between the two lines [70]. Gene expression of the two cell lines was measured with and without neutrophil differentiation treatments. Though DMSO is frequently used to solubilize compounds and act as an experimental control, it has been used to create neutrophil-like cells [71]. The validation dataset we used was generated to compare DMSO activity with untreated cells and cells treated with DMSO plus Nutridoma [33]. We tested the hypothesis that our neutrophil representation would distinguish the samples with and without neutrophil differentiation treatment. We transformed external datasets with the following operation:

$$ {W}_{k\ \mathrm{x}\ g\prime}^T\times {D}_{g\prime \mathrm{x}\ n}={D}_{k\ \mathrm{x}\ n}^{\prime } $$

where *D* represents the processed RNAseq data from GSE103706. Of 8000 genes measured in *W*, 7664 were also measured in *D* (95.8%). These 7664 genes are represented by *g’*. All of the “Neutrophils_HPCA_2” signature genes were measured in *W*. *D’* represents the GSE103706 data transformed along the specific compression feature. Each sample in *D’* is then considered transformed by the specific representation captured in *k*. The specific genes representing “Neutrophils_HPCA_2” is provided in Additional file 3.

### Downloading and processing publicly available expression data for monocyte GTEx analysis

We used an additional external dataset to validate the identified monocyte representation. We accessed processed data for the publicly available GEO dataset with accession number GSE24759 [34]. The dataset was measured by Affymetrix HG-U133A (early access array) and consisted of 211 samples representing 38 distinct and purified populations of cells, including monocytes, undergoing various stages of hematopoiesis. The samples were purified from 4 to 7 independent donors each. Many xCell gene sets were computationally derived from this dataset as well [31]. Not all genes in the weight matrices were measured in the GSE24759 dataset. For this application, 4645 genes (58.06%) corresponded with the genes used in the compression algorithms. Additionally, 168 out of 178 genes (94.38%) in the “Monocyte_FANTOM_2” gene set were measured (Additional file 3). We investigated the “Monocytes_FANTOM_2” signature because of its high enrichment in VAE *k* = 3 and low enrichment in VAE *k* = 2.

### Machine learning classification of cancer types and gene alterations in TCGA

We trained supervised learning classifiers using raw RNAseq features and BioBombe-derived features. In general, we trained supervised machine learning models to predict cancer type from RNAseq features in TCGA PanCanAtlas RNAseq data. We implemented a logistic regression classifier with an elastic net penalty. The classifiers were controlled for mutation burden. More details about the specific implementation are described in Way et al. [72]. Here, we predicted all 33 cancer types using all 11,060 samples. These predictions were independent per cancer type, which meant that we trained models with the same input gene expression or BioBombe feature data, but used 33 different status matrices.

We also trained models to predict gene alteration status in the top 50 most mutated genes in the PanCanAtlas. These models were controlled for cancer type and mutation burden. We defined the status in this task using all non-silent mutations identified with a consensus mutation caller [73]. We also considered large copy number amplifications for oncogenes and deep copy number deletions for tumor suppressor genes as previously defined [74]. We used the threshold GISTIC2.0 calls for large copy amplifications (score = 2) and deep copy deletions (score = − 2) in defining the status matrix [75]. For each gene alteration prediction, we removed samples with a hypermutator phenotype, defined by having log10 mutation counts greater than five standard deviations above the mean. For the mutation prediction task, we also did not include certain cancer types in training. We omitted cancer types if they had less than 5% or more than 95% representation of samples with the given gene alteration. The positive and negative sets must have also included at least 15 samples. We filtered out cancer types in this manner to prevent the classifiers from artificially detecting differences induced by unbalanced training sets.

We trained models with raw RNAseq data subset by the top 8000 most variably expressed genes by median absolute deviation. The training data used was the same training set used for the BioBombe procedure. We also trained models using all BioBombe compression matrices for each latent dimension and using real and permuted data. We combined compressed features together to form three different types of ensemble models. The first type grouped all five iterations of VAE models per latent dimensionality to make predictions. The second type grouped features of five different algorithms (PCA, ICA, NMF, DAE, VAE) of a single iteration together to make predictions. The third ensemble aggregated all features learned by all algorithms, all initializations, and across all latent dimensionalities, which included a total of 30,850 features. In total, considering the 33 cancer types, 50 mutations, 28 latent dimensionalities, ensemble models, raw RNAseq features, real and permuted data, and 5 initializations per compression, we trained and evaluated 32,868 different supervised models.

We optimized all of the models independently using fivefold cross validation (CV). We searched over a grid of elastic net mixing and alpha hyperparameters. The elastic net mixing parameter represents the tradeoff between l1 and l2 penalties (where mixing = 0 represents an l2 penalty) and controls the sparsity of solutions [76]. Alpha is a penalty that tunes the impact of regularization, with higher values inducing higher penalties on gene coefficients. We searched over a grid for both hyperparameters (alpha = 0.1, 0.13, 0.15, 0.2, 0.25, 0.3 and mixing = 0.15, 0.16, 0.2, 0.25, 0.3, 0.4) and selected the combination with the highest CV AUROC. For each model, we tested performance using the original held out testing set that was also used to assess compression model performance.

### Evaluating model training time

We evaluated the execution time of training each compression algorithm for all three datasets across several latent dimensionalities. We used 8 representative latent dimensionalities: *k* = 2, 4, 10, 16, 25, 50, 80, and 200. We conducted the time analysis using a CPU machine with an Intel Core i3 dual core processer with 32 GB of DDR4 memory.

### Reproducible software

All code to perform all analyses and generate all results and figures is provided with an open source license at https://github.com/greenelab/biobombe [77]. All resources can be viewed and downloaded from https://greenelab.github.io/BioBombe/.

## Supplementary information

Additional file 1. *All Supplementary Figures*. Supplementary Figures S1-S14.

Additional file 2. *Supplemental Note*. Describing neural network optimization.

Additional file 3. *Neutrophil and Monocyte Gene Sets.* Entrez gene IDs and gene symbols for two xCell gene signatures (Neutrophil_HPCA_2 and Monocyte_FANTOM_2). Associated with Fig. 6.

Additional file 4. *Model coefficients for predicting TP53 loss of function*. Using all compressed features in the model implicates compressed features with cancer hallmark signatures. Associated with Fig. 7.

Additional file 5. *Tissue types and counts for TARGET, TCGA, and GTEx*.

Additional file 6. *Hetnetpy metaedge summary.* Network summary of edge and node counts for each gene set collection.

Additional file 7. Review history.

## Abbreviations

RNAseq: RNA sequencing
PCA: Principal component analysis
ICA: Independent components analysis
NMF: Non-negative matrix factorization
AE: Autoencoder
DAE: Denoising autoencoder
VAE: Variational autoencoder
TCGA: The Cancer Genome Atlas
GTEx: Genome tissue expression project
TARGET: Therapeutically applicable research to generate effective treatments project
BRCA: Breast invasive carcinoma
COAD: Colon adenocarcinoma
LGG: Low-grade glioma
PCPG: Pheochromocytoma and paraganglioma
LAML: Acute myeloid leukemia
LUAD: Lung adenocarcinoma
GEO: Gene Expression Omnibus
ROC: Receiver operating characteristic
PR: Precision recall
AUROC: Area under the receiver operating characteristic curve
AUPR: Area under the precision recall curve
CV: Cross-validation
ORA: Overrepresentation analysis
GSEA: Gene set enrichment analysis
SVD: Singular value decomposition
CCA: Canonical correlation analysis
SVCCA: Singular vector canonical correlation analysis
TF: Transcription factor
DMSO: Dimethyl sulfoxide
