# Supplementary material for: Compressing gene expression data using multiple latent space dimensionalities learns complementary biological representations
Source: Genome Biol. 2020 May 11;21:109. doi: 10.1186/s13059-020-02021-3 (PMC7212571; doi:10.1186/s13059-020-02021-3)
Supplement: Supplementary file 2 — Supplemental Note. Describing neural network optimization. [file 13059_2020_2021_MOESM2_ESM.pdf]

Supplemental Note

Compressing gene expression data using multiple latent space dimensionalities learns complementary biological representations

Gregory P. Way[1,2,3], Michael Zietz[2], Vincent Rubinetti[2], Daniel S. Himmelstein[2] and Casey S. Greene[2*]

[1]Genomics and Computational Biology Graduate Group, Perelman School of Medicine, University of Pennsylvania, Philadelphia, PA 19104, USA.
[2]Department of Systems Pharmacology and Translational Therapeutics, University of Pennsylvania, Philadelphia, PA 19104, USA.
[3]Imaging Platform, Broad Institute of MIT and Harvard, Cambridge, MA 02142, USA.
*Corresponding Author

Optimizing Training Hyperparameters in Neural Network Architectures

We applied BioBombe using five compression algorithms. Two of the five models, variational autoencoders (VAE) and denoising autoencoders (DAE), are based on autoencoder (AE) frameworks and include several hyperparameters that must be tuned to optimize signal reconstruction. Our primary concern in training the AE models was to ameliorate potential performance biases as the bottleneck dimension increased if hyperparameters were kept static. In other words, we sought to isolate performance differences to the effects of changing k dimensionalities. Therefore, we performed a grid search around several hyperparameters for both AE models including 6 representative k dimensionalities.

Training autoencoders, and neural networks in general, requires architectural and hyperparameter decisions to optimize learning important signals in input data. We searched through a grid of various combinations of learning rates, epochs, batch sizes, sparsity, and noise parameters for DAE models, and learning rates, epochs, batch sizes, and kappa values for VAE models. We included 6 representative k dimensionalities in this grid (k = 5, 25, 50, 75, 100, and

125). While many combinations did not converge, we observed relatively consistent performance across all hyperparameter combinations tested for both AE models, especially for the VAE models (**Additional File 2: Figure S2**). We selected the hyperparameter combinations for the top performing models and used these in training downstream models.
