# Supplementary material for: Compressing gene expression data using multiple latent space dimensionalities learns complementary biological representations
Source: Genome Biol. 2020 May 11;21:109. doi: 10.1186/s13059-020-02021-3 (PMC7212571; doi:10.1186/s13059-020-02021-3)
Supplement: Supplementary file 7 — Review history. [file 13059_2020_2021_MOESM7_ESM.docx]

Review History

**First round of review**

**Reviewer 1**

**Comments to author:**

In this paper, Way et al. examine several approaches to signal extraction from RNAseq expression data. The motivation for the paper appears to be an attempt to address the question of "how many latent dimensions are really needed", but in practice this paper ends up asking (and mostly answering) the question, "is there a single best dimensionality or compression algorithm for this kind of analysis"? (The answer appears to be "no", which is fascinating.)

Briefly, the authors train PCA, ICA, NMF, denoising autoencoders, and variational autoencoders on RNAseq data from TCGA PanCanAtlas, GTEx, and TARGET. They then ask whether there are "best" latent space dimensionalities for each method, and whether the dimensions and signals extracted match across the techniques.

The answer is an emphatic "no", with a variety of fascinating observations that essentially result in a conclusion of "it's complicated." A few key takeaways that I got --

* there's no single best dimensionality for any of the techniques, although (as expected) more dimensions captures more variability

* the low dimensions do not necessarily correspond in terms of the signals they recover. While PCA/ICA/NMF seem to have strong similarity (they are all linear techniques), DAE and VAE recover distinct signals.

* recovered signals generalized to external data sets, suggesting they are intrinsic to RNAseq.

* biology is complicated and while these techniques seem to work well for certain features, we don't understand many of the features and  caution is warranted.

The paper is well written and quite clear, and serves as a nice antidote to the wide array of papers proposing that these techniques can simply be applied generically to RNAseq data to produce biological insight.  The conclusions are cautious and well supported, and internal controls (permutations!) are well done.

The paper is of broad interest because these techniques are all being used "in the wild" to understand RNAseq, by many people!

I have only one minor thoughts for improvement the language around technical artifacts could use some cohesion.  The possibility that these techniques can be used to detect technical artifacts is obvious and mentioned in several places, but the summary point is left at "we do not have a mechanism to detect them." (line 338) This bears some further discussion, space permitting, or realignment to say "we simply aren't going to look at this" at the beginning.  But the paper is fine without this change, too.

**Reviewer 2**

**Comments to author:**

In this manuscript, the authors proposed an analytic procedure, BioBombe, which runs five existing different dimension reduction methods on a gene expression data to extract latent dimensions. These five existing methods include the principal components analysis (PCA), independent components analysis (ICA), non-negative matrix factorization (NMF), denoising autoencoder (DAE), and variational autoencoder (VAE) models. BioBombe works by running each of the five methods to extract up to 200 latent dimensions and then combining all these dimensions from the five methods together. The paper applied BioBombe to perform a range of ad hoc analyses in three bulk transcriptomic databases (TCGA, GTEx, and TARGET) and picked up a few examples to show that different dimensions may sometimes correspond to different biological features. The paper eventually concluded that it is important to look at a range of different dimensionalities for different biological problems.

Dimension reduction is certainly an important analytic topic for bulk transcriptomic analysis. I also very much appreciate the authors performing a large amount of analyses across a range of biological problems. However, the proposed analytic procedure, BioBombe, lacks novelty or originality, as it simply consists of running five existing dimension reduction methods. In addition, the analyses presented in the paper lack a systematic way of results validation and appear to cherry picking results for presentation. Importantly, none of these analyses are compared to existing standard non dimension reduction approaches that are more suitable to these problems. It is thus unclear whether BioBombe can lead to new biological discoveries that cannot be achieved by standard approaches. In addition, given that dimension reduction in bulk transcriptomic analysis are often related to batch effects correction, it is surprising to see that the authors did not compare their approaches with standard batch removing methods such as SVA and PEER and did not address batch effects in BioBombe. Finally, the main conclusion from the paper that it is important to look at different dimensions for different problems is generic and vague, and is already well appreciated in the transcriptomic analysis field: practitioners almost always look at different dimensions to make sense of them at the data exploratory stage. Therefore, I am not convinced that BioBombe or the analyses presented in the paper represents a significant advance in the field. My detailed concerns are:

In the first main example (sex and MYCN amplification), BioBombe found in each case that a very high dimensional feature is associated with the variable of interest. However, I am unsure how useful it is to tell a biologist that the feature 108 in the gene expression data is associated with sex and the feature 111 is association with MYCN amplification. I am not used to seeing a mainstream biology journal reporting such a high dimension feature and then try to make sense of it. After all, if sex or MYCN amplification is an important biological feature worth investigating in the particular data set, then why would it only appear as a 100th feature instead of, say, a top 5 feature? I am thus not convinced that dimension reduction is the appropriate method to study this particular problem.

It would be important to compare BioBombe with a standard differential gene expression analysis that treats sex or MYCN amplification as the predictor variable. Unlike dimension reduction, standard differential expression analysis can pinpoint the exact set of genes that are associated with the predictor variable. In addition, to enhance interpretation or power of differential expression analysis, one can perform gene set enrichment analysis. Gene set enrichment analysis can also pinpoint the exact gene set enriched in association with the predictor variable. Compared to the standard differential expression analysis, or the standard approach of performing both differential expression analysis and gene set enrichment analysis, what would you gain by performing dimension reduction?

How do you address batch effects in dimension reduction? Do you need to first use SVA or PEER to remove confounding factors? If so, how many factors do you need to extract from SVA or PEER to represent batch effects? Since the current paper argues that high dimensional features are important, do we also need to examine up to 200 features from SVA or PEER as is done in BioBombe? In addition, how do you decide whether any feature (e.g. feature 108 or feature 111) represents the true biological signal or simply represents batch effects that also happen to be correlated with the predictor variable of interest, given that some batch effects may influence only a subset of genes and thus may appear to be a feature in the very high latent space?

In the second main example (gene set coverage), BioBombe analysis shows that a higher gene set coverage can be achieved by models with larger latent dimensionalities. I am a bit lost on the purpose of this analysis. Without seeing analysis, I would have already guessed that a large number of dimensionalities are necessary to achieve a high gene set coverage. Afterall, the feature space with the highest dimensionalities would recover the original gene expression space, which would have the highest gene set coverage. I am also surprised by the flawed definition of gene set coverage in the paper: in the paper, a gene set is considered as covered by a feature if the gene set had the highest positive or highest negative BioBombe z score compared to all other gene sets in that collection. However, this definition ignores any statistical significance level. By this definition, a gene set can be considered as covered by a feature even if it is not at all associated with the feature. Therefore, I was lost at what the paper was planning to achieve in this particular analysis. If the main purpose is to eventually use the feature space to represent or replace all pathways, then why not using pathways directly and run some gene set enrichment analyses?

In the third main example, BioBombe identified a low dimension structure (2nd or 3rd feature) that is correlated with cell types. Such batch effect presumably can also be easily and well captured by any batch removal methods. Have you tried SVA or PEER to see if the low dimensional structure from these methods also capture the cell type composition in these data? PEER is the standard batch effect removal method used in the original GTEx study. If SVA or PEER works well, then what are the benefits of running BioBombe? In addition, can the loading matrix learned by SVA or PEER applied to the external validation data set?

In the last main example of supervised learning, BioBombe can only predict cancer type as well as a simple logistic regression. However, without comparing BioBombe to some standard prediction models (e.g. lasso, elastic net, deep learning algorithms), it is hard to see that BioBombe would be useful for supervised learning. Some of these standard prediction models use the original gene space directly and are thus more interpretable than using the latent features as in BioBombe. For example, selecting a gene, say BRCA1, through a penalized regression, would naturally give much more clear biological insights to cancer, than, say, selecting feature 101 through BioBombe.

Finally, given the large number of output features (200 features from each of the five methods)), how would you recommend practitioners to use BioBombe? Do we need to manually examine through each of the 1000 latent dimensions? And what is the rational of fixing the maximum number of latent dimensionalities to be 200? Shouldn't this number be dependent on the data set (e.g. sample size, contained information)? How would you recommend us to select this number in practice? Also, what do we gain by running BioBombe compared to running each of the five existing methods used in BioBombe alone (e.g. VAE)? Why do you choose these particular five dimensionality reduction methods? It would be natural and important to explore other standard manifold learning methods such as Isomaps, locally linear embedding (LLE), etc.

Minor comments:

1. Why do you transform the gene expression values to a range between 0 and 1 before analysis? Wouldn't this lead to loss of information? Have you compared this transformation to other alternative transformation approaches? And how do you transform them into (0,1)?

2. Do you still set k=200 when the sample size is smaller than 200? In the MYCN example, how do you extract k=200 when there appears to be only 162 samples?

3. In Figure 3a, VAE produced the highest -log10 P-value with the largest k. Would this P-value decrease if you increase the dimension over 200?

4. It would be helpful to display the computation time of all the five included methods, BioBombe, as well as other competing methods in a figure or table.

5. Incomplete sentence: "... determine the 65 number of latent dimensions
66 (k) to compress the input data into" (line 64-65, page4)

6. It would be useful to create a website for BioBombe, where users can upload their own data for analysis.

**Authors Response**

**Point-by-point responses to the reviewers’ comments:**

**Reviewer #1: In this paper, Way et al. examine several approaches to signal extraction from RNAseq expression data. The motivation for the paper appears to be an attempt to address the question of "how many latent dimensions are really needed", but in practice this paper ends up asking (and mostly answering) the question, "is there a single best dimensionality or compression algorithm for this kind of analysis"? (The answer appears to be "no", which is fascinating.)**

The reviewer accurately describes our intent. We agree that the answer appears to be no. We also found this particularly intriguing, and it suggests that many of the field’s approaches to analyze “performance” in this domain may be flawed. Instead of trying to find methods that are “best”, it suggests that we would be better off trying to find methods that learn different types of biological features from existing methods, and that learning ensembles of features across methods may be the most fruitful. In the end, we had to create just such an ensemble of features to perform these evaluations. Given our findings, we also release all BioBombe models, derived gene expression features, and software as a resource to the community for further investigation and application.

Our previous title, “Sequential compression across latent space dimensions enhances gene expression signatures” focused on the *implications* of our finding but didn’t state our primary finding. To make our primary finding more direct, we changed the title of the paper to:

Sequential compression of gene expression across dimensionalities and methods reveals no single best method or dimensionality

We also note here that, in response to a comment raised by reviewer 2, we also release a website that provides curated links to all of these resources for easy viewing and download.

https://greenelab.github.io/BioBombe/

**Briefly, the authors train PCA, ICA, NMF, denoising autoencoders, and variational autoencoders on RNAseq data from TCGA PanCanAtlas, GTEx, and TARGET. They then ask whether there are "best" latent space dimensionalities for each method, and whether the dimensions and signals extracted match across the techniques.**

**The answer is an emphatic "no", with a variety of fascinating observations that essentially result in a conclusion of "it's complicated." A few key takeaways that I got --**

*** there's no single best dimensionality for any of the techniques, although (as expected) more dimensions captures more variability**

This is correct.

*** the low dimensions do not necessarily correspond in terms of the signals they recover. While PCA/ICA/NMF seem to have strong similarity (they are all linear techniques), DAE and VAE recover distinct signals.**

For PCA and ICA, we expect them to be rotations of each other, which is a nice positive control. We were somewhat surprised to see that NMF was also similar because the model assumptions are different. Our analysis suggests that the linear methods all learn similar signal. However, though the *models* capture much the same signal, the *representation* in terms of individual gene features can be distinct (for instance, in Figure 3 the Sex components of TCGA or the MYCN components of TARGET, where ICA and PCA learn features that align differently with the biological signal). We made a small revision to the discussion section to add a sentence that highlights this finding:

For example, though ICA and PCA represent rotations of each other, we found that the methods varied in their ability to capture specific biological signals into single features, which highlights the challenge of picking only a single algorithm.

*** recovered signals generalized to external data sets, suggesting they are intrinsic to RNAseq.**

This was very encouraging to us, as it suggested that the models are applicable beyond the datasets on which they were trained. We now also cite a number of recent manuscripts which further support this finding:

These features generalized to other data, providing additional evidence for the intrinsic qualities of biological representations embedded in gene expression data [1–4].

*(Note that reference numbers align with references at the end of this document and not with references in the resubmitted manuscript)*

*** biology is complicated and while these techniques seem to work well for certain features, we don't understand many of the features and caution is warranted.**

Yes - this is an important take-home message. In all of our evaluations we are considering the extent to which features can be ascribed to annotations that relate to biologically-relevant metadata variables or to which they align with known pathways.

**The paper is well written and quite clear, and serves as a nice antidote to the wide array of papers proposing that these techniques can simply be applied generically to RNAseq data to produce biological insight. The conclusions are cautious and well supported, and internal controls (permutations!) are well done.**

Thank you. We took substantial care to include methods that could serve as controls for each other (i.e., PCA and ICA) as well as to perform careful permutations to assess baseline performances.

**The paper is of broad interest because these techniques are all being used "in the wild" to understand RNAseq, by many people!**

Yes, one motivating factor for us was seeing how widespread these techniques are becoming. In our reading of the literature, we have found authors choosing a single method and single dimensionality. In recent presentations and posters at meetings, we find the same thing. It seems that because there is no systematic evaluation, folks use either what is easy to run or what their colleagues recommend.

We initially sought to understand which method was best for each benchmark dataset by selecting a large cancer dataset (TCGA), a small cancer dataset (TARGET), and a large normal tissue dataset (GTEx). However, our finding is that choosing one method to the exclusion of others may be limiting in the types of features that one can discover. Our hope is that this manuscript encourages authors to choose multiple, distinct methods across different latent space dimensionalities to generate features for downstream analyses.

**I have only one minor thoughts for improvement the language around technical artifacts could use some cohesion. The possibility that these techniques can be used to detect technical artifacts is obvious and mentioned in several places, but the summary point is left at "we do not have a mechanism to detect them." (line 338) This bears some further discussion, space permitting, or realignment to say "we simply aren't going to look at this" at the beginning. But the paper is fine without this change, too.**

We chose benchmark datasets which have already been normalized to provide a picture closest to how these are used in the wild. We now note, in the introduction, that we are seeking to understand how well these methods extract biological signal, as opposed to how well they capture technical sources of variability:

Additional latent variable approaches have been used to detect and remove technical artifacts, including batch effects [5,6]. Here, we focus on using compression algorithms to identify biological representations by analyzing processed data with batch effect already mitigated.

We also modified the sentence in the discussion that mentions this limitation and to reiterate our focus:

We also did not focus on detecting compressed features that represent technical artifacts, which has already been widely explored [8,9].

**Reviewer #2: In this manuscript, the authors proposed an analytic procedure, BioBombe, which runs five existing different dimension reduction methods on a gene expression data to extract latent dimensions. These five existing methods include the principal components analysis (PCA), independent components analysis (ICA), non-negative matrix factorization (NMF), denoising autoencoder (DAE), and variational autoencoder (VAE) models. BioBombe works by running each of the five methods to extract up to 200 latent dimensions and then combining all these dimensions from the five methods together. The paper applied BioBombe to perform a range of ad hoc analyses in three bulk transcriptomic databases (TCGA, GTEx, and TARGET) and picked up a few examples to show that different dimensions may sometimes correspond to different biological features. The paper eventually concluded that it is important to look at a range of different dimensionalities for different biological problems.**

We thank the reviewer for their summary of the manuscript. Our results suggest that different methods applied at different dimensionalities learn different types of biological representations. For example, while some biological signals are best learned at lower dimensionalities, others are optimized at higher dimensionalities. We also find that this property is different depending on the algorithm.

As part of understanding the performance characteristics of this particular set of methods we combine (1) a systematic examination and comparison across compression models with (2) a substantial dive into certain key gene expression features that compression models detect.

We felt that this most effectively characterized the similarities and differences of these methods in the context in which they are used in practice.

In order to better summarize our primary finding, and to more clearly highlight the main discovery presented in the research article, we have updated the title from “Sequential compression across latent space dimensions enhances gene expression signatures”, which described the implication of our finding but not the finding itself, to:

Sequential compression of gene expression across dimensionalities and methods reveals no single best method or dimensionality

We also specify our motivation more directly in the abstract:

In practice, most researchers select a single algorithm and latent dimensionality. We sought to determine the extent by which using multiple dimensionalities across ensemble compression models improves biological representations.

**Dimension reduction is certainly an important analytic topic for bulk transcriptomic analysis. I also very much appreciate the authors performing a large amount of analyses across a range of biological problems. However, the proposed analytic procedure, BioBombe, lacks novelty or originality, as it simply consists of running five existing dimension reduction methods. In addition, the analyses presented in the paper lack a**

**systematic way of results validation and appear to cherry picking results for presentation. Importantly, none of these analyses are compared to existing standard non dimension reduction approaches that are more suitable to these problems. It is thus unclear whether BioBombe can lead to new biological discoveries that cannot be achieved by standard approaches. In addition, given that dimension reduction in bulk transcriptomic analysis are often related to batch effects correction, it is surprising to see that the authors did not compare their approaches with standard batch removing methods such as SVA and PEER and did not address batch effects in BioBombe. Finally, the main conclusion from the paper that it is important to look at different dimensions for different problems is generic and vague, and is already well appreciated in the transcriptomic analysis field: practitioners almost always look at different dimensions to make sense of them at the data exploratory stage. Therefore, I am not convinced that BioBombe or the analyses presented in the paper represents a significant advance in the field.**

Dimensionality reduction is becoming increasingly and widely used, and we appreciate the reviewer’s view that the topic is of high importance. Our goal with this manuscript was to understand the performance characteristics of methods that are commonly applied to compressing gene expression data. In particular, we sought to understand whether or not there was a compression method or a particular latent space dimensionality that dominated others in terms of its ability to recover biological representations. We assessed performance through numerous evaluations. Our evaluations included the examination of individual features associated with key biological endpoints as well as systematic comparisons across a large number of endpoints. The systematic comparisons included:

1. In the stability and model similarity analysis, we compared compressed representations

across model initializations, across different algorithms, and across latent dimensionalities for TCGA, GTEx and TARGET (Figure 2, Supplementary Figure S4, and Supplementary Figure S5). We showed that the linear methods (PCA, ICA, NMF) are stable across initializations and largely identify similar signals. The autoencoder methods (DAE, VAE) were less stable and identified different biological representations. 2. We calculated gene set coverage across algorithms and latent space dimensionalities. In

this analysis, we concluded that using ensemble models across different latent dimensionalities maximizes biological representations in different gene sets (Figure 4). NMF models identified the fewest number of gene sets, but found them with high enrichment. VAE and DAE models identified the most unique genesets. 3. We observed that features generated by the different compression algorithms at different latent space dimensionalities can be used to predict cancer type and mutation status in The Cancer Genome Atlas (Figure 7). Cancer type is a feature typically learned in lower dimensionalities than certain molecular features. We also observed that features learned by different algorithms and at different dimensionalities all contributed to prediction tasks, which further highlights the importance of using ensemble methods across many latent dimensionalities for optimizing biological representations.

We also agree that the compression models applied are not novel - all have been effectively applied to extract biological signals from bulk gene expression data in previous studies. However, the novelty is in using the sequential compression approach, where we vary the

internal compression dimensionalities, to systematically compare the extracted signals across commonly applied methods. As reviewer 1 noted above, the paper provides an “antidote” to the one-model one-dimensionality approach to signal extraction. For practitioners who already test multiple algorithm conditions, this manuscript provides evidence of what is lost by using and reporting only a one-model one-dimensionality result.

Based on the comments above, it is apparent that we did not effectively convey the primary finding. We have made a series of changes to improve clarity.

We have revised the title to be:

Sequential compression of gene expression across dimensionalities and methods reveals no single best method or dimensionality

and the conclusions section of the abstract to state:

There is no single best latent dimensionality or compression algorithm for analyzing gene expression data. Instead, using feature ensembles from different compression models across latent space dimensionalities optimizes biological representations.

We reiterate our primary findings in the background section:

Our primary finding is that there is no single algorithm or dimensionality that is best for all purposes: Instead, using various latent dimensionalities and algorithms optimizes biological representations. Those who plan to apply these algorithms may consider training multiple models over multiple dimensionalities to avoid missing important biological features.

We add to the introduction specific motivation to why we selected the specific algorithms to study:

We selected these methods because they are either widely established in practice (PCA, NMF) or use neural networks that are rapidly growing in popularity (DAE, VAE). We included ICA to compare against PCA: it has been used in practice [7] and also allows us to compare multiple rotations of the low-dimensional space. We applied these methods to three different datasets...

We add a statement in the background section describing our motivation for focusing on extracting biological signals and not technical sources of variation including batch effect.

Additional latent variable approaches have been used to detect and remove technical artifacts, including batch effects [5,6]. Here, we focus on using compression algorithms to identify biological representations by analyzing processed data with batch effect already mitigated.

We add a sentence in the discussion section to reiterate our focus:

We also did not focus on detecting compressed features that represent technical artifacts, which has already been widely explored [8,9].

Lastly, throughout the entire manuscript we tighten language surrounding our primary motivation. *Our goal is not to introduce BioBombe as a new method. Instead, we use BioBombe as a means to understand low-dimensional representations of gene expression via the models constructed and through an ensemble of compression methods across several latent space dimensionalities.* We have attempted to clarify this in the revision.

*(Note that reference numbers align with references at the end of this document and not with references in the resubmitted manuscript)*

**My detailed concerns are:**

**In the first main example (sex and MYCN amplification), BioBombe found in each case that a very high dimensional feature is associated with the variable of interest. However, I am unsure how useful it is to tell a biologist that the feature 108 in the gene expression data is associated with sex and the feature 111 is association with MYCN amplification. I am not used to seeing a mainstream biology journal reporting such a high dimension feature and then try to make sense of it. After all, if sex or MYCN amplification is an important biological feature worth investigating in the particular data set, then why would it only appear as a 100th feature instead of, say, a top 5 feature? I am thus not convinced that dimension reduction is the appropriate method to study this particular problem.**

Though journals rarely report a specific feature to highlight, features that are not successfully extracted are invisible for any downstream analysis. If that analysis is to select principal components that capture 90% of the variance in the data before performing visualization (e.g. t- SNE) of samples, or to calculate distance in a reduced dimensional space, or anything else: if it wasn’t captured, it won’t be found. Sex, or more specifically, gene expression signatures that are associated with X-inactivation and genes on the Y chromosome, forms a well-known signal in gene expression data. Therefore, this signal serves as a universal positive control in gene expression datasets that includes males and females. Methods should be able to extract this signal if provided with sufficient dimensionality. We find that beyond 50 dimensions in GTEx, the signal becomes apparent. Though sex is not within the top 5 features of gene expression data, the feature is certainly important.

We also sought to evaluate important biological endpoints that are less-well understood than sex. MYCN amplification is a nice example of one of these. It’s a biologically important feature of neuroblastoma and it has important implications for the outcome of children with this tumor type. Tumors with MYCN amplification are aggressive and associated with poor outcome. Exploring this feature also provides insights into how methods perform: past the twelfth principal component the signal never changes and the signal actually decays in ICA models; however, for NMF, DAE, and VAE models the clarity of the signal increases throughout the range of dimensionalities that we evaluated.

Thus, though we don’t expect that people would usually report a specific component in a high- dimensional model, understanding what that model does and more importantly does not capture

remains relevant to any downstream use of the reduced dimensional space that it defines. We previously used BioBombe as a shorthand for the set of features included in the model.

We have now revised the introduction of this section to clarify that we’re not testing a method (BioBombe) but instead using the features that comprise the BioBombe collection to understand the methods:

We tested the ability of features derived using the BioBombe approach to isolate various biological representations.

And, we clarify the approach in the methods section that introduces this analysis.

We tested BioBombe sequentially compressed features to distinguish sample sex in GTEx and TCGA data, and MYCN amplification in TARGET NBL data. We tested all compression algorithms and latent space dimensionalities to determine the conditions in which these features were best captured.

We also provide similar language when referencing BioBombe features throughout the manuscript to maintain consistency. We update the subheading of this section to more clearly state our motivation and primary finding:

BioBombe features capture various gene expression representations across different latent dimensionalities

We also update the subsection heading in the methods section to:

Assessing gene expression signatures present in BioBombe features

**It would be important to compare BioBombe with a standard differential gene expression analysis that treats sex or MYCN amplification as the predictor variable. Unlike dimension reduction, standard differential expression analysis can pinpoint the exact set of genes that are associated with the predictor variable. In addition, to enhance interpretation or power of differential expression analysis, one can perform gene set enrichment analysis. Gene set enrichment analysis can also pinpoint the exact gene set enriched in association with the predictor variable. Compared to the standard differential expression analysis, or the standard approach of performing both differential expression analysis and gene set enrichment analysis, what would you gain by performing dimension reduction?**

Our goal was to understand the collection of methods at various dimensionalities because these reduced dimensional spaces are increasingly used to provide an embedding of high-dimensional data for downstream analyses. A differential expression analysis would have told us what we already know, namely that genes that differentiate sex are those encoded on the Y chromosome, those involved in X inactivation, or specific hormone regulation, but would not have illuminated the latent dimensionalities at which these features are captured or the performance characteristics of the methods used to derive the features that comprise the BioBombe set.

**How do you address batch effects in dimension reduction? Do you need to first use SVA or PEER to remove confounding factors? If so, how many factors do you need to extract from SVA or PEER to represent batch effects? Since the current paper argues that high dimensional features are important, do we also need to examine up to 200 features from SVA or PEER as is done in BioBombe? In addition, how do you decide whether any feature (e.g. feature 108 or feature 111) represents the true biological signal or simply represents batch effects that also happen to be correlated with the predictor variable of interest, given that some batch effects may influence only a subset of genes and thus may appear to be a feature in the very high latent space?**

We would expect researchers to use standard randomization strategies during experimental design and to remove batch effects before downstream analysis. We used large benchmark datasets for which these steps had already been performed to maximize our ability to examine the performance characteristics of the methods. Still, we expect that not all batch effects may be removed and that they may appear as features in a high-dimensional latent space. This is one reason we focus our initial examination on the well-understood feature of sex.

A reasonable approach to determine whether the highest scoring feature represents sex is to look at the genes that contribute to that feature with high weight. Therefore, we used the Sex Associated Gene Database (SAGD) [10] to map compressed feature gene weights to known sex differences across genes.

Comparing feature weights for GTEx NMF feature 111 k = 200 (x axis; left) to the negative log10 p value of sex differences across genes according to SAGD (y axis), we can see how three genes with extremely significant sex differences dominate this feature. These three genes are all encoded on the Y chromosome (*RPS4Y1*, *KDM5D*, and *DDX3Y*).

Furthermore, performing the same comparison for TCGA (TCGA ICA feature 151 k = 200; right), we observe that the top 10 genes are all genes encoded on the X chromosome. This indicates that we are extracting sex specific genes in the TCGA analysis as well.

In summary, our examination of these feature reveals that the algorithms are identifying sex in TCGA and GTEx. This result further provides evidence that this feature does not represent a batch effect. We have now added this to the results:

We examined the genes that contribute with high weight to this feature and found only three genes had substantial influence. These three genes all had high positive weights and were encoded on the Y chromosome.

The separation was not as strong using the more complex TCGA data, but the top 10 gene weights were all encoded on the X chromosome.

*Additional note regarding the gene expression signature analysis and results (in response to the broader points raised by reviewer 2)*

We understand the confusion of this specific analysis, especially in the context of a manuscript where the primary goal was to compare different compression methods and latent space dimensionalities. Based on the way this section was originally written, we agree that it sounds like we are presenting a new method. We’ve revised the entire section to be more reflective of an investigation of signatures from the context of comparing methods and latent space dimensionalities.

We also thank the reviewer for bringing up these specific and detailed concerns. Not only did it enable us to more specifically focus the manuscript (and this section in particular), but the concerns also directed us towards a better controlled signature analysis approach. Specifically, we dove deeper into the specific genes with high weights in the weight matrices that the signature analysis identified. We used the Sex Associated Gene Database [10] to map compressed feature gene weights to known sex differences across genes.

We also updated the analysis to include only sample types with balanced representation across tissue type (in GTEx) and primary tissue sites (TCGA). We also included only primary neuroblastoma samples in the TARGET analysis. We did this to reduce the impact of metadata variables enriched in certain classes. For example, we removed all breast and prostate cancer samples from the TCGA analysis. In addition, we removed the equal variance assumption in all t-tests. Taken together, these updates changed the features with the greatest observed p value for all comparisons. For example, by removing the equal variance assumption, the approach revealed an NMF feature that isolated male samples in GTEx. Additionally, the new highest feature in TCGA was identified in an ICA model with a latent dimensionality of 90 and the new highest feature in TARGET was identified in a VAE model with latent dimensionality of 50.

We also added t-test statistics for all boxplot comparisons to this figure (GTEx sex, TCGA sex, MYCN amplification within Neuroblastoma tumors only, and MYCN amplification in a validation set)

We update the methods section to reflect this change:

Using the sample activation scores for each of the top scoring features for sample sex in TCGA and GTEx, and MYCN amplification in TARGET and the validation set, we performed two

tailed t-test with unequal variance comparing each group. For the TCGA and GTEx sex comparison, our t-test compared male vs. female activation scores. For the TARGET and NBL cell line analyses, our t-test compared MYCN amplified NBL samples vs. MYCN non-amplified NBL samples. We add t-test statistics and p values in each sub figure.

and provide the updated figure below:

Note: All of the specific software changes introduced in our response to the gene signature analysis can be viewed in the following pull requests:

<https://github.com/greenelab/BioBombe/pull/184> <https://github.com/greenelab/BioBombe/pull/186>

**In the second main example (gene set coverage), BioBombe analysis shows that a higher gene set coverage can be achieved by models with larger latent dimensionalities. I am a bit lost on the purpose of this analysis. Without seeing analysis, I would have already guessed that a large number of dimensionalities are necessary to achieve a high gene set coverage. Afterall, the feature space with the highest dimensionalities would recover the original gene expression space, which would have the highest gene set coverage. I am also surprised by the flawed definition of gene set coverage in the paper: in the paper, a gene set is considered as covered by a feature if the gene set had the highest positive or highest negative BioBombe z score compared to all other gene sets in that collection. However, this definition ignores any statistical significance level. By this definition, a gene set can be considered as covered by a feature even if it is not at all associated with the feature.**

We also would guess that more features would be associated with higher gene set coverage for exactly the same reason. However, there are a number of results in this section that were not obvious in advance of the analyses:

1. The linear methods (ICA, PCA, NMF) all “covered” the most gene sets. 2. The autoencoder methods had the highest enrichment for the sets that they covered. 3. The autoencoder methods contributed the most unique gene sets. 4. While models compressed with larger latent space dimensions had higher gene set

coverage, many individual gene sets were captured with the highest enrichment in models with low and intermediate dimensions.

We also did not ignore statistical significance. As the manuscript notes: “We removed gene sets from consideration if their p values were not lower than a Bonferroni adjusted value determined by the total number of latent dimensionalities in the model.” This means that large models are penalized more than smaller ones. However, this sentence used to be in the middle of a paragraph of the methods section and may have been overlooked. We have added a paragraph break immediately after the sentence to draw attention to this step:

We removed gene sets from consideration if their p values were not lower than a Bonferroni adjusted value determined by the total number of latent dimensionalities in the model.

We calculated coverage (C) by considering all unique top gene sets (U) identified...

We also add specific mention of how gene sets are assigned to BioBombe derived features in the results:

Briefly, we projected all compressed features onto a gene set network and assigned gene sets with the highest enrichment that passed an adjusted statistical significance threshold to each compressed feature (see methods for more details).

**Therefore, I was lost at what the paper was planning to achieve in this particular analysis. If the main purpose is to eventually use the feature space to represent or replace all pathways, then why not using pathways directly and run some gene set enrichment analyses?**

Our goal was to understand the performance of individual methods as well as the full collection of features from the aggregation of the individual methods. If we had used the pathways directly we would not have been able to evaluate these dimensionality reduction approaches.

We were interested in determining at which latent dimensionalities specific features were found with the highest enrichment. Therefore, to highlight this primary finding, we revised the final sentence of this section to now read:

These results did not reveal a best method or dimensionality: Various biological representations are best discovered by using various compression algorithms with various latent space dimensionalities.

**In the third main example, BioBombe identified a low dimension structure (2nd or 3rd feature) that is correlated with cell types. Such batch effect presumably can also be easily and well captured by any batch removal methods. Have you tried SVA or PEER to see if the low dimensional structure from these methods also capture the cell type composition in these data? PEER is the standard batch effect removal method used in the original GTEx study. If SVA or PEER works well, then what are the benefits of running BioBombe? In addition, can the loading matrix learned by SVA or PEER applied to the external validation data set?**

We had two goals in examining low dimensional structure of the data in this analysis:

● We sought to understand method performance in this space because this is a context where compression is often applied. Users of these methods who select a modest number of principal components or low dimensionalities will end up in this space. It is important to understand what they do and do not capture.

● Examining very low dimensional structure could have revealed clear and obvious batch effects of the type usually removed with SVA or PEER. Following the corrections performed by the originators of the data, our analyses did not indicate obvious batch effects.

BioBombe helped us to understand the performance characteristics of these methods. In this specific analysis, it revealed that at low dimensionalities the models can capture quite different features at the same dimensionality. We found the result where different tissue signal was captured in different orders across methods striking (Figure 5d). Based on this finding we more deeply probed the result, in large part to understand if the result appeared to be biological or artefactual, which was why we looked more deeply into cell types.

In summary, these analyses revealed non-obvious properties of the methods to us. Examining the results suggested that the increased capture of blood cell signal could be attributed to biologically-relevant processes, suggesting that it is not driven by artifacts.

We more specifically describe our motivation for this analysis in the introductory sentence of the section introducing these results.

To determine if this rapid increase was a result of models learning specific biological representations or if this observation represented a technical artifact, we more closely examined the sharp increase in GTEx blood tissue correlation between latent space dimensionalities 2 and 3 in VAE models...

**In the last main example of supervised learning, BioBombe can only predict cancer type as well as a simple logistic regression. However, without comparing BioBombe to some standard prediction models (e.g. lasso, elastic net, deep learning algorithms), it is hard to see that BioBombe would be useful for supervised learning. Some of these standard prediction models use the original gene space directly and are thus more interpretable than using the latent features as in BioBombe. For example, selecting a gene, say BRCA1, through a penalized regression, would naturally give much more clear biological insights to cancer, than, say, selecting feature 101 through BioBombe.**

This systematic evaluation was designed to measure the extent to which each compression method captures biologically-important features at various latent space dimensionalities. BioBombe is not being used as a supervised learning approach, but instead, we input BioBombe derived features into standard prediction models (logistic regression with elastic net penalty) in a supervised learning framework. The ability of the BioBombe derived features (from different algorithms and different latent dimensionalities) to predict cancer-type or mutation status tells us how well (and at what dimensionality) the features are capturing signal aligned with these biological endpoints. We agree that this point was confusing in the methods section, so we add a sentence to the beginning of the methods subsection where this analysis is introduced:

We trained supervised learning classifiers using RNA-seq features and BioBombe-derived features.

We compare these methods against a negative control (permuted data) and positive control (raw gene expression data). We expect that performance should generally exceed that of permuted data and be lower than that of the raw gene expression data. This is what we observe. We also included a biological negative control, Titan (TTN), which is a large gene and so among the most frequently mutated genes in cancer but which is expected to have no effect (passenger mutations). This is what we observe. We included this large number of controls so that we could better understand method performance and biases. However, within those bounds were a number of interesting findings:

● An ensemble of models exceeded the performance of individual models.

● Denoising autoencoders could generate very predictive features but the run-to-run variability between these models is quite high.

● The VAE, in general, provided the most efficient compression for capturing features that predicted mutation status or cancer type.

● Cancer types are frequently captured at lower dimensionality than mutations, but for each method there is an overlap region where the strongest mutation features are captured before all of the cancer type signal is.

**Finally, given the large number of output features (200 features from each of the five methods)), how would you recommend practitioners to use BioBombe? Do we need to manually examine through each of the 1000 latent dimensions? And what is the rational of fixing the maximum number of latent dimensionalities to be 200? Shouldn't this number be dependent on the data set (e.g. sample size, contained information)? How would you recommend us to select this number in practice? Also, what do we gain by running BioBombe compared to running each of the five existing methods used in BioBombe alone (e.g. VAE)? Why do you choose these particular five dimensionality reduction methods? It would be natural and important to explore other standard manifold learning methods such as Isomaps, locally linear embedding (LLE), etc.**

We are generating a total of 185,100 features: we use three datasets, five algorithms, randomly permuted data, 28 different latent dimensionalities ranging from k = 2 to k = 200, and five different initializations for each. The paper systematically compares the features generated in each context.

In practice, the selected latent space dimensionality should be dependent on the dataset. The rationale was that computing becomes increasingly expensive as the model size grows, and this was the level that we could feasibly complete across this systematic comparison of algorithms and datasets for a reasonable number of replicates.

BioBombe reveals relationships among these methods as well as the extent to which methods capture biological features. Running one of the methods that comprises the BioBombe collection will capture some signal from the dataset; however, in each evaluation in this paper we found that different methods have different strengths and weaknesses. If compute time is a challenge, then we would recommend that users pick one linear method along with the VAE. The VAE was more stable than the DAE, and the VAE and linear methods are most divergent in terms of their performance characteristics. We chose methods are broadly established (PCA, NMF), rapidly growing in practice (DAE, VAE), or those that served as important internal controls (ICA). We now clarify this in the introduction:

We selected these methods because they are either widely established in practice (PCA, NMF) or use neural networks that are rapidly growing in popularity (DAE, VAE). We included ICA to compare against PCA: it has been used in practice [7] and also allows us to compare multiple rotations of the low-dimensional space. We applied these methods to three different datasets.

We also update the conclusions subsection of the abstract to more specifically state how users should apply compression models to extract biological representations:

There is no single best latent dimensionality or compression algorithm for analyzing gene expression data. Instead, using feature ensembles from different compression models across latent space dimensionalities optimizes biological representations.

We also add our recommendation to users in the background section.

Our primary finding is that there is no single algorithm or dimensionality that is best for all purposes: Instead, using various latent dimensionalities and algorithms optimizes biological representations. Those who plan to apply these algorithms may consider training multiple models over multiple dimensionalities to avoid missing important biological features.

**Minor comments:**

**1. Why do you transform the gene expression values to a range between 0 and 1 before analysis? Wouldn't this lead to loss of information? Have you compared this transformation to other alternative transformation approaches? And how do you transform them into (0,1)?**

Feature normalization avoids over-weighting features (e.g. genes) that have a particularly large range, and is important for algorithms with reconstruction metrics. In this case, we considered a more standard conversion to z-scores; however, NMF requires a positive matrix. We linearly transformed input features to [0,1], which can be used with each of the algorithms, so we selected it to provide the same input matrix to each algorithm. We now note this.

We selected this range because it was compatible with all of the algorithms.

We are also more descriptive in the methods about how this transform is performed.

Before input into the compression algorithms, we transformed the gene expression values by gene to the [0, 1] range by subtracting the minimum value and dividing by the range for each specific gene. We applied this transform independently for the testing and training partitions.

**2. Do you still set k=200 when the sample size is smaller than 200? In the MYCN example, how do you extract k=200 when there appears to be only 162 samples?**

The TARGET data have 734 samples, which we used for training. A subset of the TARGET data are assays of neuroblastoma samples. MYCN amplification is important for neuroblastoma. We trained the models on the full dataset, and then we evaluated the extent to which methods were able to capture this feature within the neuroblastoma subset of samples. We previously said: “Using latent features derived from the full TARGET data, we performed a two-tailed t-test comparing MYCN amplified vs. MYCN not amplified NBL tumors.” We have now revised the sentence to put more emphasis on the full set of samples being used:

We performed a two-tailed t-test comparing MYCN amplified vs. MYCN not amplified NBL tumors for each of the latent features derived from the full set of TARGET samples.

**3. In Figure 3a, VAE produced the highest -log10 P-value with the largest k. Would this P- value decrease if you increase the dimension over 200?**

It is always hard to guess what would happen without testing it. Our supposition is that it would continue to increase to some point, at which point it would either level off or then decrease. However, without the results we cannot know this for sure.

**4. It would be helpful to display the computation time of all the five included methods, BioBombe, as well as other competing methods in a figure or table.**

We agree that this would be helpful. We have added figure comparing compute time across compression methods and different latent dimensions for each dataset.

We add reference to this figure in the discussion and add the figure below:

First, our approach takes a long time to run. We are training many different algorithms across many different latent dimensions and iterations, which requires a lot of compute time (**Additional File 1: Figure S14**).

The specific changes that introduce this analysis can be viewed here:

https://github.com/greenelab/BioBombe/pull/185

We also add details of this analysis to the methods section:

*Evaluating Model Training Time*

We evaluated the execution time of training each compression algorithm for all three datasets across several latent dimensionalities. We used 8 representative latent dimensionalities: k = 2, 4, 10, 16, 25, 50, 80, and 200. We conducted the time analysis using a CPU machine with an Intel Core i3 dual core processer with 32 GB of DDR4 memory.

**5. Incomplete sentence: "... determine the 65 number of latent dimensions 66 (k) to compress the input data into" (line 64-65, page4)**

We thank the reviewer for pointing this out, we reworded the sentence to avoid ending with a preposition:

Nevertheless, a major challenge to all compression applications is the fundamental requirement that a researcher must determine the number of latent dimensions (k) into which they wish to compress the input data.

**6. It would be useful to create a website for BioBombe, where users can upload their own data for analysis.**

We see the BioBombe approach as a detailed examination of the performance characteristics of a set of compression algorithms, applied at various latent dimensionalities, across benchmark datasets. We have provided source code for the approach, and we have distributed each of the models that comprise the BioBombe collection. We have also been contacted by a few users of the BioBombe models and source code with feedback. We expect that the users of the resource will be primarily interested in either using the source code or in downloading the collection of models. We agree that there are many outputs, and we have prepared a website that links out to archived versions for each of the resources.

The website can be viewed here:

<https://greenelab.github.io/BioBomb/>

References:

1. Dincer AB, Celik S, Hiranuma N, Lee S-I. DeepProfile: Deep learning of cancer molecular profiles for precision medicine. bioRxiv [Internet]. 2018 [cited 2019 Sep 17]; Available from: http://biorxiv.org/lookup/doi/10.1101/278739

2. Taroni JN, Grayson PC, Hu Q, Eddy S, Kretzler M, Merkel PA, et al. MultiPLIER: a transfer learning framework reveals systemic features of rare autoimmune disease. 2018 [cited 2018 Sep 9]; Available from: http://biorxiv.org/lookup/doi/10.1101/395947

3. Wang J, Agarwal D, Huang M, Hu G, Zhou Z, Ye C, et al. Data denoising with transfer learning in single-cell transcriptomics. Nat Methods. 2019;16:875–8.

4. Stein-O’Brien GL, Clark BS, Sherman T, Zibetti C, Hu Q, Sealfon R, et al. Decomposing Cell Identity for Transfer Learning across Cellular Measurements, Platforms, Tissues, and Species. Cell Syst. 2019;8:395–411.e8.

5. Johnson WE, Li C, Rabinovic A. Adjusting batch effects in microarray expression data using empirical Bayes methods. Biostatistics. 2007;8:118–27.

6. Stegle O, Parts L, Piipari M, Winn J, Durbin R. Using probabilistic estimation of expression residuals (PEER) to obtain increased power and interpretability of gene expression analyses. Nat Protoc. 2012;7:500–7.

7. Zhou W, Altman RB. Data-driven human transcriptomic modules determined by independent component analysis. BMC Bioinformatics [Internet]. 2018 [cited 2018 Dec 22];19. Available from: https://bmcbioinformatics.biomedcentral.com/articles/10.1186/s12859-018-2338-4

8. Leek JT, Storey JD. Capturing Heterogeneity in Gene Expression Studies by Surrogate Variable Analysis. PLoS Genet. 2007;3:e161.

9. Teschendorff AE, Zhuang J, Widschwendter M. Independent surrogate variable analysis to deconvolve confounding factors in large-scale microarray profiling studies. Bioinformatics. 2011;27:1496–505.

10. Shi M-W, Zhang N-A, Shi C-P, Liu C-J, Luo Z-H, Wang D-Y, et al. SAGD: a comprehensive sex-associated gene database from transcriptomes. Nucleic Acids Res. 2019;47:D835–40.

**Second round of review**

**Reviewer 2**

Thank you for the detailed reply to my previous comments. I don’t think my previous main comments were well addressed, but this is likely due to me not clearing stating my precious concerns precisely and concisely. Let me organize my previous comments into a single main point/concern here. To begin with, there are two potential messages the paper might intend to convey: (1) lower latent dimensionalities contain biological information and thus would be useful to look at; (2) lower latent dimensionalities are more likely to contain biological information or likely contain more biological information than higher latent dimensionalities. From my understanding of the paper, I believe the authors only intend to cover the first message, even though the paper reads as if it is also attempting to cover the second message; therefore, some further clarifications at key places of the paper could be helpful. As I brought up in my comments earlier, the first message is a guaranteed mathematical property that is well recognized by analysts in the field. I am not aware of anyone or any previous publications that would deny that first message – if so, citing these previous publications would certainly help strengthening the paper.

However, my previous main concern was that the first message lacks practical importance as the lower latent dimensionalities typically contain biological information that are less important than the higher latent dimensionalities. Subsequently, it is challenging to justify spending resources examining lower latent dimensionalities, while these precious recourses could be spent on examining higher latent dimensionalities or examining other more suitable analytic approaches other than dimension reduction that could lead to much more important biological findings. For example, in my previous first main comment, I mentioned that sex was only the 108th feature and MYCN is only the 111th feature, and thus it was unclear how to justify looking at these two features while there were ~100 features above them. I am not saying that SEX/MYCN is not an important biological feature; I am just saying that SEX/MYCN does not appear to be an important biological feature for these particular data sets as there are ~100 other features above SEX/MYCN. Indeed, as you showed in your reply, the sex feature is only associated with 3 Y chr genes and does not contain the key sex gene XIST. Therefore, the practical question is, how do you justify to spend resource to look at this 108 or 111 feature, instead of spending resources to first look at the top ~100 features or spending resources to perform other analysis (e.g. DE analysis, which would guarantee to identify the most important sex gene XIST). These later two analyses are what analysts would typically do in practice: examining higher latent dimensionality would allow you to identify truly important biological features in the particular data; performing DE analysis would help identify genes associated with sex if sex is your main biological feature. Comparing to these two standard approaches is thus critical for demonstrating the practical importance of the first message. My main concern that the first message lacks practical importance is also directly linked to my other previous main comment: given the large number of output features (185,000 features) extracted by BioBombe, how would you recommend us to use BioBombe? Do we need to manually examine through each of these 185,000 latent dimensions – if so, how to justify it given that many standard non dimension reduction approaches exist and would be more suitable for the particular biology question you asked in all these examples (e.g. DE analysis for the first couple examples; gene set enrichment analysis in a middle example; or lasso/elasticnet type prediction methods for the cancer type example)? I guess overall I still have trouble understanding how BioBombe would help us to improve practical data analysis.

**Reviewer 3**

I tend to side with Reviewer 2 here, in that it is rather hard to understand how Biobombe is meant to be used, practically speaking. The compelling example of sex being component 108 (but actually only a bit of sex -- 3 genes not including XIST!) is a good one. I worry that this is a bit the failing of dimension reduction .. sex is spread out across many components -- XIST is surely in there somewhere.

The analysis and understanding of what you get from dimensionality reduction is still interesting and if the authors could give a clear answer "how BioBombe would help us to improve practical data analysis" (maybe they already did?), that would be still quite interesting.

The authors also say they operate on "processed data with batch effect already mitigated" .. but that doesn't seem to be the point at which such a method would most often be applied. At least in most of our collaborative work, the dimension reduction is used as a way to get understand batch (though we often work in smaller datasets ..). The authors completely remove this from the equation.

**Authors Response**

**Point-by-point responses to the reviewers’ comments:**

Reviewer #2: Thank you for the detailed reply to my previous comments. I don’t think my previous main comments were well addressed, but this is likely due to me not clearing stating my precious concerns precisely and concisely. Let me organize my previous comments into a single main point/concern here.

To begin with, there are two potential messages the paper might intend to convey: (1) lower latent dimensionalities contain biological information and thus would be useful to look at; (2) lower latent dimensionalities are more likely to contain biological information or likely contain more biological information than higher latent dimensionalities. From my understanding of the paper, I believe the authors only intend to cover the first message, even though the paper reads as if it is also attempting to cover the second message; therefore, some further clarifications at key places of the paper could be helpful.

We thank the reviewer for elaborating on their main concern. Our message is not explicitly linked to “lower latent dimensionalities” (we address this potential point of confusion later on in this response). Instead, our message is that different compression models trained with different latent dimensionalities **will learn different representations.** We describe these differences with a series of comprehensive analyses, which we complement with vignettes characterizing certain biological features. We have revised the abstract more precisely on this primary message, which builds clarity on changes that were introduced after the first round of review. The refined abstract now states:

In practice, most researchers fit a single algorithm and latent dimensionality. We sought to determine the extent to which selecting only one fit limits the biological features captured in the latent representations and, consequently, limits what can be discovered with subsequent analyses.

We have also now captured our primary finding in a new, declarative title that emphasizes the research finding:

“Compressing gene expression data using multiple latent space dimensionalities learns complementary biological representations”

We recognize that among our findings, the primary implications of the results were not clearly communicated. In response, we have capitalized on the reviewer’s concern and have made substantial changes to the language describing the motivation, background, and results of each analysis. We provide all of our changes in the attached manuscript (changes tracked in Microsoft Word). We also list every major change below:

1. We’ve completely revamped the abstract’s results subsection to now state:

We compressed gene expression data from three large datasets consisting of adult normal tissue, adult cancer tissue, and pediatric cancer tissue. We trained many different models across a large range of latent space dimensionalities and observed various performance differences. We identified more curated pathway gene sets significantly associated with individual dimensions in denoising autoencoder and variational autoencoder models trained using an intermediate number of latent dimensionalities. Combining compressed features across algorithms and dimensionalities captured the most pathway-associated representations. When trained with different latent dimensionalities, models learn strongly-associated and generalizable biological representations; including sex, neuroblastoma MYCN amplification, and cell-types. Stronger signals, such as tumor type, were best captured in models trained at lower dimensionalities, while more subtle signals, such as pathway activity, were best identified in models trained with more latent dimensionalities.

2. We’ve explicitly clarified the breakdown of the paper to better manage expectations

and avoid confusion regarding our primary motivation. This section replaces the third paragraph in the Background section and now states:

The paper is divided into two parts. First, we describe model performance in different contexts. We observed differences in reconstruction cost, stability, and gene set coverage across datasets, algorithms, and latent dimensionalities. Second, we present a series of vignettes highlighting differences in biological representations driven by the number of latent dimensionalities used during model training. We observed that distinct gene expression representations are best captured in different models spanning low, intermediate, and high latent dimensionalities. Our primary finding is that there is no single algorithm or dimensionality that is best for all purposes: Instead, using various latent dimensionalities and algorithms optimizes biological representations. Researchers who plan to apply these algorithms to gene expression data should consider training multiple models over multiple latent dimensionalities to optimize and avoid missing important biological representations.

We also add a sentence to introduce the first part of the manuscript (model performance):

In the first part of the paper, we report specific and commonly applied performance metrics for all algorithms and latent dimensionalities.

And we add an introductory sentence to results subsection 5, which introduces the second part of the manuscript (describing specific biological representations):

In the second part of the paper, we tested the ability of different latent space dimensionalities and algorithms to capture various biological signals. We first tested the ability of all BioBombe features to differentiate common and well-characterized biological representations.

We also change this subsection heading to read:

Different latent space dimensionalities and algorithms capture specific gene expression representations at variable resolution

3. We add a brief introduction to the section that describes gene set coverage. We feel

that this addition better captures our motivation for this analysis:

The BioBombe approach generates many different features associated with various biological representations. As part of our rigorous evaluation, we generated 30,850 features per dataset. The features are generated in an unsupervised fashion, and, in order to maximize utility, they require interpretation. One interpretation approach involves projecting gene weights onto existing biological networks (see Fig 1a). This approach not only calculates enrichment scores of specific biological gene sets and pathways for individual BioBombe features, but also enables us to track how these enrichment scores evolve across latent dimensionalities, and to quantify the percentage of characterized gene sets in each collection. We define this percentage as “gene set coverage".

We also change this subsection heading:

Large-scale interpretation of BioBombe compressed features: Assessing gene set coverage

As I brought up in my comments earlier, the first message is a guaranteed mathematical property that is well recognized by analysts in the field. I am not aware of anyone or any previous publications that would deny that first message – if so, citing these previous publications would certainly help strengthening the paper.

However, my previous main concern was that the first message lacks practical importance as the lower latent dimensionalities typically contain biological information that are less important than the higher latent dimensionalities. Subsequently, it is challenging to justify spending resources examining lower latent dimensionalities, while these precious recourses could be spent on examining higher latent dimensionalities or examining other more suitable analytic approaches other than dimension reduction that could lead to much more important biological findings. For example, in my previous first main comment, I mentioned that sex was only the 108th feature and MYCN is only the 111th feature, and thus it was unclear how to justify looking at these two features while there were ~100 features above them. I am not saying that SEX/MYCN is not an important biological feature; I am just saying that SEX/MYCN does not appear to be an important biological feature for these particular data sets as there are ~100 other features above SEX/MYCN.

Indeed, as you showed in your reply, the sex feature is only associated with 3 Y chr genes and does not contain the key sex gene XIST. Therefore, the practical question is, how do you justify to spend resource to look at this 108 or 111 feature, instead of spending resources to first look at the top ~100 features or spending resources to perform other analysis (e.g. DE analysis, which would guarantee to identify the most important sex gene XIST).

We thank the reviewer for providing their specific thoughts on this issue. This comment may result from a common misconception about how dimensionality reduction algorithms identify latent space features. While it is certainly true that principle components analysis (PCA) will identify deterministic and orthogonal features with decreasing amounts of variance explained [1], **it is incorrect to apply this logic to the other dimensionality reduction algorithms we analyzed**. Independent components analysis (ICA), non-negative matrix factorization (NMF), denoising autoencoders (DAE), and variational autoencoders (VAE) are all stochastic and will identify different representations assigned to a different component number (e.g. PCA component 1) and depending on the number of latent dimensionalities the user selects [2].

To empirically demonstrate this property, we performed a simulation experiment. In this experiment, we artificially injected two distinct signals (each represented by a block of three correlated features) into a simulated dataset. The first group of signals had a higher correlation structure than the second group (see figure left). Using this covariance structure, we randomly sampled 10,000 samples. The complete simulated dataset consisted of 10,000 samples and 10 features. 4 of these features were sampled with random Gaussian noise. We applied a

BioBombe approach (PCA, ICA, NMF, DAE, and VAE with k = 1, 2, 3, 4, 5, and 6) to these data. We visualized the top compressed features that captured each correlated feature (features 1, 2, and 3 in block 1 and 5, 6, and 7 in block 2) with the highest weight. All compression algorithms were able to aggregate the two groups of signals together into the same feature (with some exceptions, see figures below).

PCA identified both groups of features with a deterministic solution and decreasing signal strength independent of the latent dimensionality. **This was not the case for every other algorithm in the simulation**.

The k=6 row provides an example. The first correlated block of simulated features emerges in PC1, while the second emerges in PC2. However, in ICA the first block of simulated features emerges in IC3, while the second emerges in IC5. This is because ICA does not have the same feature importance / compressed feature number relationship that PCA does. The NMF model behaves similarly to PCA in this context because of the simple covariance structure (we observe, in our broader study, that NMF does not always have this property in practice for gene expression data). The DAE learns a representation that mixes the signals across three dimensions. The VAE learns a representation where the second correlated block of features is captured in VAE3, but the first is separated into VAE2 and VAE6. Code to reproduce this analysis is introduced in the BioBombe repository in this pull request: https://github.com/greenelab/BioBombe/pull/188

Further evidence of this effect can be seen in the existing figures. For example, in figure 6c and 6d (reproduced below), we observe interesting patterns across algorithms:

Specifically, in both c) and d), the PCA models (red) identify both gene sets (neutrophils and monocytes) once at k=6. This score does not change in PCA models because the models are deterministic. It is also safe to interpret that, in PCA models, a biological feature associated with Neutrophils and Monocytes provides the 6^th^ most variance. However, the patterns in all other algorithms are quite different. VAE and DAE models capture neutrophils at low dimensionalities, and they fluctuate with high scores throughout the remaining latent dimensionalities. In contrast, all other algorithms learn about Monocytes roughly around k=6. However, while ICA models maximize their score between k=6 and k=12, their ability to capture monocyte signals actually degrades in models trained at higher latent dimensionalities. The opposite is true for NMF models, where their performance actually increases throughout the k range, and especially in high capacity models (k>100). **The observation that certain signatures degrade is a direct example of why it is not safe to conclude that large models capture, in individual features, every biological dimension that smaller models can.**

We have now clarified these points in the text:

1. In the second paragraph of the background section, where we first introduce the

compression algorithms we used in the study, we add:

Specifically, we trained PCA, ICA, NMF, DAE, and VAE models using three different gene expression datasets. We selected these methods because they are either widely established in practice (PCA, ICA, NMF) or use neural networks that are rapidly growing in popularity (DAE, VAE). Furthermore, it is well known that PCA will identify a unique and deterministic solution that represents compressed features with a decreasing amount of variance explained [1]. However, the other models do not share this property. In these other models, different latent space dimensionalities and model initializations will identify different feature representations, and the feature number has no inherent ordering [2].

2. In the 3rd results subsection (stability and similarity using singular vector canonical

correlation analysis (SVCCA)) where we compare results across algorithms. This result *also* adds further support to the idea that different latent dimensionalities and algorithms identify different biological representations. We now add:

We also used SVCCA to compare the similarity of weight matrices extracted from models trained with different latent dimensionalities. Both PCA and ICA found highly similar solutions (Fig. 2b). This is expected since PCA solutions are deterministic and are arranged with decreasing amounts of variance, and ICA is a rotation of PCA space. We do not observe these patterns for the other compression algorithms.

3. In the 6th results subsection (tissue and cell-type composition analysis), we add a

section that further interprets Fig 6c and 6d.

Fig. 6c and Fig. 6d provide concrete examples of differences across algorithm and latent space dimensionalities. Specifically, PCA identifies both neutrophil and monocyte gene sets at *k* = 6, and then does not identify any other feature with more signal. This is expected since PCA solutions are deterministic and ordered by decreasing variance explained. However, we do not observe this pattern in other algorithms. The AE models capture the neutrophil signature early, and then fluctuate in isolating its signal (Fig. 6c). Furthermore, while all algorithms capture monocytes around *k* = 6, NMF increases signal capture in larger latent dimensionality models and ICA decreases signal capture. Lastly, PCA captures this monocyte gene set as well as other algorithms for most dimensionalities, but the opposite is true for the neutrophil gene set. A researcher selecting only a single compression model at one latent space dimensionality might clearly observe certain features while others could be obscured.

These later two analyses are what analysts would typically do in practice: examining higher latent dimensionality would allow you to identify truly important biological features in the particular data; performing DE analysis would help identify genes associated with sex if sex is your main biological feature. Comparing to these two standard approaches is thus critical for demonstrating the practical importance of the first message.

We agree precisely with this important point. In standard practice, if a researcher is interested in identifying genes associated with a specific phenotype (e.g. sex, or MYCN amplification), the correct approach is to apply a differential expression analysis. We are not proposing BioBombe as an alternative to differential expression analysis. We are using these phenotypes because 1) they are signals well known to be present in gene expression data, 2) we can examine the behavior of each algorithm and latent space dimensionality in capturing their signal. We hypothesized that different latent space dimensionalities and different algorithms will capture this signal to varying degrees. This is indeed what we observed.

To make this important distinction clearer, we have updated the results subsection that introduces the analysis:

In the second part of the paper, we tested the ability of different latent space dimensionalities and algorithms to capture various biological signals. We first tested the ability of all BioBombe features to differentiate common and well-characterized biological representations. We describe the ability of BioBombe features to isolate sample sex; which has been previously observed to be captured in latent space features [3–5].

We also summarize our finding that both algorithm and dimensionality affect what is captured in each representation at the end of this results subsection:

Taken together, these analyses demonstrate that different compression models best identify specific biological representations when trained with different latent space dimensionalities.

Because this is a crucial point, we also make it clear in the discussion section where we discuss approach limitations:

Furthermore, the BioBombe approach is not a replacement for differential gene expression analysis in implicating genes associated with a specific phenotype. For example, if a researcher’s goal was to identify all genes contributing to sex differences or MYCN amplification, then they would apply a differential expression analysis. We used these vignettes to demonstrate trade-offs in how well different algorithms and latent dimensionalities capture these signals.

My main concern that the first message lacks practical importance is also directly linked to my other previous main comment: given the large number of output features (185,000 features) extracted by BioBombe, how would you recommend us to use BioBombe? Do we need to manually examine through each of these 185,000 latent dimensions – if so, how to justify it given that many standard non dimension reduction approaches exist and would be more suitable for the particular biology question you asked in all these examples (e.g. DE analysis for the first couple examples; gene set enrichment analysis in a middle example; or lasso/elasticnet type prediction methods for the cancer type example)? I guess overall I still have trouble understanding how BioBombe would help us to improve practical data analysis.

We thank the reviewer for summarizing their concerns. We believe that our responses to the previous comments have made our analysis and justification clear: If a user selects only one algorithm and one latent dimensionality they risk failure to identify many biological features. Furthermore, a single one of these algorithms is often applied in the scope of a larger analytical process (e.g., before visualization) and missing features will be eliminated in all subsequent steps as well. This makes it critically important to understand how using only a single algorithm and dimensionality limits downstream findings. As we noted in the prior response, the large number of dimensions enabled us to rigorously test these algorithms over multiple datasets.

There is an additional (and separate) important point made in the reviewer comment above: How does one use BioBombe? First, our primary goal is to provide rationale for researchers to switch paradigms and collect biological representations from multiple algorithms and latent space dimensionalities,

**if the goal is discovering novel biology**. We add clarity to this distinction throughout the revision (including updating the title and major abstract revisions). Indeed, the article is being submitted as a “research” article and not a “method” or “software” article. Second, in the manuscript, we did provide one approach to use BioBombe: We project compressed features (gene weights) onto gene set networks and compare gene set activations against randomized networks (Figure 1 below demonstrates this approach).

Though our primary goal was to describe the results of our research into algorithms and dimensionalities, it is now clear that this approach can be applied more generally. Since we posted this article as a preprint, this network projection approach has already been used by a different group (Palla and Ferrero) [6]. Pertinent text from the Palla and Ferrero preprint is:

Gene set coverage score was computed by means of heterogeneous networks, as described in Way et al. Briefly, we made use of heterogeneous network made available by Way et al. or generated as part of this study from several gene set collections (MSigDB52, KEGG, REACTOME, Biocarta, MetaBase (Clarivate Analytics MetaBase® version 6.15.62452), WikiPathways). We also generated respective shuffled networks in order to calculate a Z- score for each gene set – latent variable pair. We then converted the Z-score to a p-value and filtered using a Bonferroni correction (p-value threshold defined as 0.01 divided by the number of latent variables for the specific model – latent space dimensionality pair). For each gene set collection and each latent variable, the top gene set was selected to be mapped to that latent variable.

To make this point clear in the text, we add text introducing this section. We believe that this text links together the purpose of BioBombe as a “research” article, while proposing network projection as a quick way to interpret compressed features.

The BioBombe approach generates many different features associated with various biological representations. In our example, we generated 30,850 features per dataset. The features are generated in an unsupervised fashion, and, in order to maximize utility, they require interpretation. One interpretation approach involves projecting gene weights onto existing biological networks (see Fig 1a). This approach not only calculates enrichment scores of specific biological gene sets

and pathways for individual BioBombe features, but also enables us to track how these enrichment scores evolve across latent dimensionalities, and to quantify the percentage of characterized gene sets in each collection. We define this percentage as “gene set coverage".

We also add the following sentence to the discussion:

Here, we present a novel option to rapidly interpret compressed features based on network- projection [7,8].

Reviewer #3: I tend to side with Reviewer 2 here, in that it is rather hard to understand how Biobombe is meant to be used, practically speaking. The compelling example of sex being component 108 (but actually only a bit of sex -- 3 genes not including XIST!) is a good one. I worry that this is a bit the failing of dimension reduction .. sex is spread out across many components -- XIST is surely in there somewhere.

We thank the reviewer for pointing out this important observation. We believe that the primary motivation for this analysis was not clear. Indeed, it is in fact a “failing of dimensionality reduction” if the primary goal of the analysis was to identify sex-specific genes. We agree that an alternative approach would be more appropriate. However, this was not our goal. Instead, our goal was to observe how sex signal is captured by different dimensionality reduction algorithms across different latent space dimensionalities. To make this important distinction clear, we add:

While this analysis demonstrates that sex is identified with varying signal strength across algorithms and latent dimensionalities, it also highlights that compression algorithms do not completely capture all genes that differentiate sex into a single feature. To identify genes with expression that varies by sex, it would be best to apply a differential expression analysis [9,10].

In addition, we stop using the term “signature” when discussing BioBombe features, and instead use the term “representation”. We feel that the term “signature” more strongly implies a comprehensive list of upregulated and downregulated genes, while the term “representation” allows for varying degrees of signal. We make this change throughout the manuscript, with perhaps the most noticeable change happening in the figure legend of Figure 3. Instead of reading “using BioBombe as a signature discovery tool”, we now state:

Exploring the ability of BioBombe features across algorithms and latent dimensionalities to detect biological representations.

Lastly, we make it clear in the discussion section that a differential expression analysis approach is the preferred way to form a comprehensive signature for certain biological signal:

Furthermore, the BioBombe approach is not a replacement for differential gene expression analysis in implicating genes associated with a specific phenotype. For example, if a scientist’s goal was to identify all genes contributing to sex differences or MYCN amplification, then they would apply a differential expression analysis. We used these vignettes to demonstrate trade- offs in how well different algorithms and latent dimensionalities capture these signals.

The analysis and understanding of what you get from dimensionality reduction is still interesting and if the authors could give a clear answer "how BioBombe would help us to improve practical data analysis", that would be still quite interesting.

We thank the reviewer for their interest in this approach. Indeed, we agree that, after our revisions to improve clarity, the primary message will help researchers to improve practical data analysis. The primary message is now more clearly reflected in the title:

“Compressing gene expression data using multiple latent space dimensionalities learns complementary biological representations”

We also add an important perspective in the results subsection of the abstract, that will remind the reviewer of our primary message:

We compressed gene expression data from three large datasets consisting of adult normal tissue, adult cancer tissue, and pediatric cancer tissue. We trained many different models across a large range of latent space dimensionalities and observed various performance differences. We identified more curated pathway gene sets significantly associated with individual dimensions in denoising autoencoder and variational autoencoder models trained using an intermediate number of latent dimensionalities. Combining compressed features across algorithms and dimensionalities captured the most pathway-associated representations. When trained with different latent dimensionalities, models learn strongly associated and generalizable biological representations; including sex, neuroblastoma MYCN amplification, and cell-types. Stronger signals, such as tumor type, were best captured in models trained at lower dimensionalities, while more subtle signals, such as pathway activity, were best identified in models trained with more latent dimensionalities.

As we noted in response to Reviewer 1, there is now a preprint that repurposes the biobombe interpretation strategy from another research group. Palla and Ferrero write:

Gene set coverage score was computed by means of heterogeneous networks, as described in Way et al. Briefly, we made use of heterogeneous network made available by Way et al. or generated as part of this study from several gene set collections (MSigDB52, KEGG, REACTOME, Biocarta, MetaBase (Clarivate Analytics MetaBase® version 6.15.62452), WikiPathways). We also generated respective shuffled networks in order to calculate a Z- score for each gene set – latent variable pair. We then converted the Z-score to a p-value and filtered using a Bonferroni correction (p-value threshold defined as 0.01 divided by the number of latent variables for the specific model – latent space dimensionality pair). For each gene set collection and each latent variable, the top gene set was selected to be mapped to that latent variable.

The authors also say they operate on "processed data with batch effect already mitigated" .. but that doesn't seem to be the point at which such a method would most often be applied. At least in most of our collaborative work, the dimension reduction is used as a way to get understand batch (though we often work in smaller datasets ..). The authors completely remove this from the equation.

We agree that dimensionality reduction algorithms are also quite helpful when used to identify sources of technical variation, including batch effect. These algorithms also have an alternative use, which is the focus of the manuscript. There is a growing body of work representing a rapidly evolving field of using unsupervised machine learning models to extract knowledge from gene expression data (we’ve written a recent review discussing this in more detail [11]). Our focus in the present manuscript is for this use. Specifically, we highlight the ability of dimensionality reduction algorithms to identify more biological representations when using multiple models across multiple latent space dimensionalities. As reviewer 1 in the first set of reviews succinctly summarized: “[The paper] serves as a nice antidote to the wide array of papers proposing that these techniques can simply be applied generically to RNAseq data to produce biological insight.”

References: 1. Jolliffe IT, Cadima J. Principal component analysis: a review and recent developments. Philos Transact A Math Phys Eng Sci. 2016;374:20150202.

2. Kairov U, Cantini L, Greco A, Molkenov A, Czerwinska U, Barillot E, et al. Determining the optimal number of independent components for reproducible transcriptomic data analysis. BMC Genomics. 2017;18:712.

3. Way GP, Greene CS. Extracting a biologically relevant latent space from cancer transcriptomes with variational autoencoders. Pac Symp Biocomput Pac Symp Biocomput. 2018;23:80–91.

4. Clark B, Stein-O’Brien G, Shiau F, Cannon G, Davis E, Sherman T, et al. Comprehensive analysis of retinal development at single cell resolution identifies NFI factors as essential for mitotic exit and specification of late-born cells. bioRxiv [Internet]. 2018 [cited 2019 Feb 17];https://doi.org/10.1101/378950. Available from: http://biorxiv.org/lookup/doi/10.1101/378950

5. Stein-O’Brien GL, Clark BS, Sherman T, Zibetti C, Hu Q, Sealfon R, et al. Decomposing Cell Identity for Transfer Learning across Cellular Measurements, Platforms, Tissues, and Species. Cell Syst. 2019;8:395–411.e8.

6. Palla G, Ferrero E. Latent factor modelling of scRNA-seq data uncovers novel pathways dysregulated in cell subsets of autoimmune disease patients. bioRxiv. 2019;

7. Fang Z, Tian W, Ji H. A network-based gene-weighting approach for pathway analysis. Cell Res. 2012;22:565–80.

8. Dong X, Hao Y, Wang X, Tian W. LEGO: a novel method for gene set over-representation analysis by incorporating network-based gene weights. Sci Rep [Internet]. 2016 [cited 2019 Jan 14];6. Available from: http://www.nature.com/articles/srep18871

9. Gershoni M, Pietrokovski S. The landscape of sex-differential transcriptome and its consequent selection in human adults. BMC Biol [Internet]. 2017 [cited 2020 Jan 25];15. Available from: http://bmcbiol.biomedcentral.com/articles/10.1186/s12915-017-0352-z

10. Shi M-W, Zhang N-A, Shi C-P, Liu C-J, Luo Z-H, Wang D-Y, et al. SAGD: a comprehensive sex- associated gene database from transcriptomes. Nucleic Acids Res. 2019;47:D835–40.

11. Way GP, Greene CS. Discovering Pathway and Cell Type Signatures in Transcriptomic Compendia with Machine Learning. Annu Rev Biomed Data Sci. 2019;2:1–17.
